# Supplementary material for: Impacts of human mobility on the citywide transmission dynamics of 18 respiratory viruses in pre- and post-COVID-19 pandemic years
Source: Nat Commun. 2024 May 16;15:4164. doi: 10.1038/s41467-024-48528-2 (PMC11098821; doi:10.1038/s41467-024-48528-2)
Supplement: Supplementary file 1 — Supplementary Information [file 41467_2024_48528_MOESM1_ESM.pdf]

Supplementary Information for

Impacts of human mobility on the citywide transmission dynamics of 18 respiratory viruses in pre- and post-COVID-19 pandemic years

Amanda C. Perofsky*, Chelsea L. Hansen, Roy Burstein, Shanda Boyle, Robin Prentice, Cooper Marshall, David Reinhart, Ben Capodanno, Melissa Truong, Kristen Schwabe-Fry, Kayla Kuchta, Brian Pfau, Zack Acker, Jover Lee, Thomas R. Sibley, Evan McDermot, Leslie Rodriguez-Salas, Jeremy Stone, Luis Gamboa, Peter D. Han, Amanda Adler, Alpana Waghmare, Michael L. Jackson, Mike Famulare, Jay Shendure, Trevor Bedford, Helen Y. Chu, Janet A. Englund, Lea M. Starita, Cécile Viboud

*Corresponding author: Amanda C. Perofsky, acperof@uw.edu

Table of Contents

Supplementary Methods ***2***

Seattle Flu Study surveillance arms **2**

Syndromic surveillance data **3**

Statistical Analysis **4**

Reporting delays 4

Transmission modeling 4

Multivariable generalized additive regression models 6

Short-term forecasting of daily transmissibility 6

Supplementary Results ***8***

Short-term forecasts of rhinovirus (hRV), adenovirus (AdV), and SARS-CoV-2 transmission **8**

Supplementary Figures ***9***

Supplementary Tables ***37***

Supplementary References ***45***

Supplementary Methods

Seattle Flu Study surveillance arms

Recruitment sites and sample sizes are listed in Table S2.

Community and clinic kiosks. In the first year of SFS, participants with acute respiratory illness (ARI) were recruited at stand-alone kiosks in 7 clinical facilities (emergency departments, clinic and urgent care waiting rooms) and at 14 public sites, including the UW campus, SeaTac airport, workplaces, and high-traffic tourist areas. Participants were eligible to enroll if they had two or more new or worsening respiratory symptoms in the previous 7 days (fever, cough, sore throat, headache, diarrhea, nausea, or vomiting, runny or stuffy nose, rash, fatigue, muscle or body aches, increased trouble with breathing, and/or ear pain or discharge) and were English- or Spanish-speaking. After completing a brief screening for eligibility to participate, individuals were consented. Upon enrolling, participants (or parent/guardian for minors) completed a questionnaire to collect participant demographics, illness characteristics, and behavioral and other clinical data. Trained research staff collected middle turbinate swabs for respiratory virus testing (Copan Diagnostics Inc., Murietta, CA). Participants received a $10 gift card for completing the study, and no additional study-related follow-up occurred. Participants were not permitted to re-enroll within a 7-day period.

Outpatient clinics (Kaiser Permanente). From November 2018 to March 2020, participants seeking outpatient care for acute respiratory illness (ARI) at Seattle-based US Flu Vaccine Effectiveness (VE) Network sites were prospectively identified and recruited through Kaiser Permanente as part of the CDC Flu VE surveillance protocol[1,2]. Patients eligible for the CDC Flu VE study were aged at least 6 months of age and had a cough illness of < 8 days duration. Eligible and consenting patients (or parent/guardian for minors) were interviewed for demographics, risk factors for ARI, and influenza vaccination history. Study staff collected combined nasal and oropharyngeal swabs (nasal only in children aged < 2 years) for respiratory virus testing. In accordance with UW IRB approval, Health Insurance Portability and Accountability Act (HIPAA) authorization and written, informed consent was waived, as there was no direct contact with these participants or reasonable ability to recontact them for consent to participate in the study. Samples were obtained through a contractual agreement with Kaiser Permanente and transported to the study laboratory at the University of Washington (UW) for further molecular testing.

Swab-and-Send Study. From October 2019 to March 2020, SFS deployed swab-and-send kits to collect nasal swabs from individuals in the community experiencing ARI. Study design, recruitment, and data collection are described in detail elsewhere[3]. Briefly, study participants were recruited through referrals from health care providers, clinics, SFS community kiosks, schools, and workplaces, dissemination of printed flyers posted at community locations, and social media advertising. Individuals were eligible to participate in the study if they lived within the greater Seattle region, had experienced new or worsening cough and/or two ARI symptoms (fever, headache, sore throat or itchy/scratchy throat, nausea or vomiting, runny/stuffy nose or sneezing, fatigue, muscle or body aches, increased trouble with breathing, diarrhea, ear pain/discharge, or rash) within 7 days of enrollment, were English speaking, had a valid email address, and access to the Internet at home. After an initial online screening questionnaire and consenting to participate in the research study, eligible participants completed an online enrollment questionnaire to provide their home address and contact information. Enrollees were mailed a home sample collection kit within 48 hours via private courier. Upon kit receipt, participants completed an online illness questionnaire to collect demographics, illness characteristics, and data on health behaviors. Samples were self-collected by participants 13 years and older via unsupervised middle turbinate swab (Copan Diagnostics Inc.). Parents or guardians performed swab collection for children younger than 13 years. Pediatric nasal swabs (Copan Diagnostics Inc.) were available for participants 5 years of age or younger. Participants were encouraged to return their nasal specimen within 24 hours or as soon as possible. Swab samples were returned to the study laboratory at UW via USPS Priority Mail prepaid postage, with a median time of 3 days from nasal swab collection to receipt at the lab[3].

Greater Seattle Coronavirus Assessment Network. The greater Seattle Coronavirus Assessment Network (SCAN) was launched on March 23, 2020, and concluded in July 2022. Design, recruitment, and data collection for SCAN are described in detail elsewhere[4,5]. Briefly, SCAN was restricted to King County, WA residents and recruited participants through social media advertising and community outreach. Eligibility criteria changed over time in response to testing demand and and were based on Public Use Microdata Areas (PUMA) and reported symptoms. Each PUMA had a daily allocation of enrollments, with over sampling of PUMAs in southern King County to

ensure more equitable access to testing across the county population[4,5]. Study materials were available in English and 12 of the most spoken non-English languages in King County. Although symptom quotas changed over time, the majority of participants were symptomatic at the time of enrollment (> 90%)[5], with symptomatic enrollees defined as individuals who self-reported experiencing a new or worsening fever, cough, or shortness of breath within the past 7 days, and asymptomatic enrollees defined as individuals self-reporting none of these symptoms. In addition to community enrollments, some participants were invited as part of Public Health – Seattle & King County (PHSKC) contact tracing efforts or through collaborations with community-based organizations to increase testing of underrepresented or high-risk populations; these samples were excluded from the analysis. After an initial online screening questionnaire, eligible participants (or parent/guardian for minors) were prompted to complete a detailed demographic and health behavior questionnaire. Within 24 hours of enrollment, sample collection kits were delivered via private courier. Samples were self-collected by participants aged 13 years and older via unsupervised middle turbinate or anterior nares swabs. Parents or guardians performed swab collection for children younger than 13 years of age. Swab samples were picked up by private courier on the morning after delivery and returned within 24 hours to the study laboratory at UW for testing.

King County COVID-19 drive-through testing sites. Beginning in April 2021, SFS obtained residual nasal swab specimens collected at eight Public Health – Seattle & King County (PHSKC) COVID-19 drive-through testing sites. In accordance with UW IRB approval, HIPAA authorization and written, informed consent was waived, as there was no direct contact with these participants or reasonable ability to recontact them for consent to participate in the study. Samples were obtained through a contractual agreement with UW Virology, which conducted the SARS-CoV-2 testing for PHSKC drive-through sites. At the time of testing, individuals completed an optional questionnaire that collected demographics, the reason for testing, COVID-19 vaccination status, whether they are currently symptomatic, and, if symptomatic, the number of days since symptom onset. Samples from both symptomatic and asymptomatic individuals were obtained by SFS, with symptomatic individuals defined as those who answered "yes" to the question "Do you have COVID-19 symptoms now?"

Residual hospital samples. Since the inception of the study, SFS obtained residual nasal swab specimens collected at clinician discretion from major hospitals in the Seattle area, including Seattle Children's, UW Medical Center, Northwest Hospital, and Harborview Medical Center. In April 2020, surveillance from UW Medical Center and Northwest Hospital discontinued. Samples were linked with demographic and clinical metadata extracted from the patients' electronic medical records (EMR). In accordance with UW IRB approval, HIPAA authorization and written, informed consent was waived, as there was no direct contact with these participants or reasonable ability to recontact them for consent to participate in the study. Samples were obtained through contractual agreements with each medical center and transported to the study laboratory at UW for further molecular testing. Encounter IDs and medical record numbers (MRNs) were used in combination as unique patient identifiers at sites except Seattle Children's, where a unique patient ID was created. Prior to March 2020, most hospital residuals were collected from patients experiencing ARI. After March 2020, there was increased testing of asymptomatic individuals at hospitals due to pre-procedure or surveillance testing for COVID-19. We used International Classification of Diseases, Tenth Revision **(**ICD-10) codes specific to respiratory illness (Harborview Medical Center, Northwest Hospital, and UW Medical Center) (Table S8) or pre-procedure COVID-19 testing flags (Seattle Children's) to distinguish symptomatic and asymptomatic patients.

Syndromic surveillance data

We obtained respiratory syndromic surveillance data for King County, WA from the Rapid Health Information Network (RHINO) program at the Washington Department of Health (WA DOH) (Fig. S23). Syndrome criteria are defined by the Electronic Surveillance System for the Early Notification of Community-Based Epidemics (ESSENCE). We received weekly counts of total emergency department (ED) visits and ED visits classified as *influenza-like illness* (ILI) (mention OR diagnosis of influenza OR fever ($\geq$100°F) and cough OR fever ($\geq$100F) and sore throat), *COVID-like illness* (CLI) (mention OR diagnosis of coronavirus AND no diagnosis of influenza OR fever OR chills AND cough OR shortness of breath OR difficulty breathing), and *broad respiratory illness* (acute bronchitis OR chest congestion OR cough OR difficulty breathing OR hemoptysis OR laryngitis OR lower respiratory infection OR nasal congestion OR otitis media OR pneumonia OR shortness of breath OR sore throat OR upper respiratory infection OR wheezing OR acute respiratory distress). Weekly data were disaggregated by age group (0-4, 5-24, 25-64, and ≥65). We collapsed age groups into two categories, < 5 and ≥ 5 years of age, and calculated the weekly proportion of ED visits coded as ILI, CLI, or broad respiratory illness (Fig. S23).

Respiratory syndromic surveillance data for Washington state were obtained from the U.S. Outpatient Influenza-like Illness Surveillance Network (ILINet) via the CDC FluView Interactive dashboard (cdcfluview R package)[6,7]. ILINet consists of approximately 3,200 sentinel outpatient healthcare providers throughout the United States that report the total number of consultations for any reason and the number of consultations for ILI every week. ILI is defined as fever (temperature of 100°F [37.8°C] or greater) and a cough and/or a sore throat. ILINet provides the weekly proportion of outpatient consultations for ILI and the number of ILI encounters by age group (0-4, 5-24, 25-64, and ≥65). We collapsed age groups into two categories, < 5 and ≥ 5 years of age, and calculated the weekly proportion of outpatient visits for ILI for each age group.

Statistical Analysis

Reporting delays

We estimated reporting delays (i.e., the delay from symptom onset to testing) using questionnaire metadata collected from symptomatic individuals who tested positive for endemic respiratory viruses (hRV, N = 4848 survey responses; EV, N = 376 ; influenza viruses, N = 830; RSV, N = 423; hPIV, N = 325; hCoV, N = 666; hMPV, N = 148; AdV, N = 443) or SARS-CoV-2 (N = 3566). We used the probabilistic programming language Stan[8] to fit a lognormal distribution to 100 subsampled bootstraps (each with 250 samples drawn with replacement) of the available reporting delay data for endemic virus and SARS-CoV-2 infections, with a maximum allowed delay of 30 days (EpiNow2 R package[9]). This resulted in a lognormal onset-to-testing delay distribution with mean 0.49 (1.02 SD) days for endemic viruses and mean 0.65 (1.1 SD) days for SARS-CoV-2.

Transmission modeling

For each pathogen, we estimated time-varying (instantaneous) reproduction numbers, R_t, by date of infection using the Epidemia R package[10,11]. Epidemia implements semi-mechanistic Bayesian epidemiological models using Stan[8].

Model specifications.

Formally, R_t is modelled as:

$$R_t = \exp(\beta_o + \epsilon_t^1), \tag{1}$$

$$\beta_o \sim \text{Normal}(\log(R_o), 0.2), \tag{2}$$

$$\epsilon_t^1 \sim \text{Normal}(0, \sigma_\epsilon), \tag{3}$$

$$\sigma_\epsilon \sim \text{Half} - \text{Normal}(0, 0.02) \tag{4}$$

where exp is the exponential function, the mean of the prior for the intercept β_o is the natural log of the basic reproduction number R_o (Table S7), and ϵ_t^1 is a daily random walk process. The steps of the daily walks ϵ_t^1 are independent and centered around 0 with standard deviation σ_ϵ.

Instead of using a renewal process to propagate infections, we modelled new infections i_t as unknown latent parameters i_t', because the additional variance around infections can account for uncertainty in initial growth rates, as well as superspreading events[10,11]:

$$i_t \sim \text{Normal}(i_t', d), \tag{5}$$

$$d \sim \text{Normal}(10, 2), \tag{6}$$

where d is the coefficient of dispersion. This prior assumes that infections have conditional variance around 10 times the conditional mean[10].

For each pathogen, we specified a generation interval distribution g_k, which is the probability that s days separate the moment of infection in an index case and in an offspring case. The intrinsic generation interval is rarely observable, but it can be approximated with the serial interval (the time between symptom onsets)[12]. We obtained pathogen-specific generation or serial intervals from published literature (Table S7).

Given the generation (or serial) interval distribution g_k, the number of new infections on day *t* is given by the convolution function:

$$i'_t = R_t \sum_{s<t} i_s g_{t-s}, \tag{7}$$

where R_t is the non-negative instantaneous reproduction number. R_t can be expressed as the number of new infections on day *t* relative to the cumulative sum of individuals infected *s* days before day *t*, weighted by the current infectiousness of those individuals[12,13]:

$$R_t = \frac{i'_t}{\sum_{s<t} i_s g_{t-s}} \tag{8}$$

The model is initialized with seeded infections $i_{v:0}$, $v < 0$, which are treated as unknown parameters[25,26]. The prior on $i_{v:0}$ assumes that daily seeds are constant over a seeding period of 6 days:

$$i_{-6:0} \sim \text{Exponential}(\tau^{-1}), \tag{9}$$

$$\tau \sim \text{Exponential}(\lambda_0), \tag{10}$$

where $\lambda_0 > 0$ is a rate hyperparameter. λ_0 is given an uninformative prior (0.03) so that seeds are primarily determined by initial transmission rates and the chosen start date of the epidemic[25,26].

Daily case counts Y_t are modelled as deriving from past new infections $i_s, s < t$, assuming a negative binomial observation model with mean y_t and overdispersion parameter ϕ. The expected number of observed cases at time *t* was mapped to past infections by convolving over the time distribution of infection to case observation π_k:

$$Y_t \sim \text{NegativeBinomial}(y_t, \phi) \tag{11}$$

$$\phi \sim \text{Normal}(10,5) \tag{12}$$

$$\text{logit}(y_t) = \sum_{s \le t} i_s \pi_{t-s} \tag{13}$$

For each pathogen, we estimated π_k by summing the incubation period distribution and the reporting delay distribution. We obtained incubation periods from published literature (Table S7) and estimated reporting delays from individual-level surveillance data, as described in the *Reporting Delays* section.

Prior to R_t estimation, we computed proxies of daily case counts of endemic pathogens by multiplying reconstructed incidences by 1000 and rounding the resultant values to integers. To reduce the influence of day-of-week effects and observational noise, we smoothed endemic pathogen case counts with centered 3-week moving averages prior to deconvolution[13,14]. Epidemic trajectories were fit independently using Stan's Hamiltonian Monte Carlo sampler[15]. For each model, we ran 4 chains, each for 30,000 iterations (including a burn-in period of 15,000 iterations that was discarded), producing a total posterior sample size of 60,000. We verified convergence by confirming that all parameters had sufficiently low R-hat values (all R-hat < 1.1) and sufficiently large effective sample sizes (>15% of the total sample size). Models were fit using the high-performance computational resources of the Biowulf Linux cluster at the National Institutes of Health, with R version 4.2.0.

Multivariable generalized additive regression models

For each pathogen, we used generalized additive models (GAMs) to measure non-linear relationships between mobility and R_t and to assess the relative importance of different behavioral indicators in predicting R_t during key epidemiological timepoints. These time periods included the 2019-2020 respiratory virus season, prior to the COVID-19 pandemic (September 2019 – February 2020), the first three months of each of four COVID-19 waves, the first six months of rebound of non-enveloped viruses (June – November 2020), the first three months of rebound of each enveloped virus in 2021, and the decline of endemic viruses during the Omicron wave in late 2021 (November 2021 – January 2022). To reduce the confounding effects of susceptible depletion on R_t, GAMs were fit to the exponential growth phase of each outbreak[16], when R_t exceeds 1 and susceptible depletion is limited.

GAMs are nonparametric regression models that replace parametric terms with (smooth) nonparametric functions of the covariates. This approach allows relationships between covariates and the dependent variable to be linear or nonlinear, depending on underlying patterns in the data. We used the `gam()` function in the mgcv R package[17] to fit each GAM with a Gamma error distribution and log link. Mobility covariates and time trends were modelled using thin plate regression splines (the default smoothing basis in mgcv). We specified for the model to add an extra penalty to each term so that it could be penalized to zero (`select = TRUE`), which enables the smoothing parameter estimation to completely remove terms when fitting the model.

To further refine our set of predictors and reduce concurvity, we used Akaike's Information Criteria corrected for small sample sizes (AICc) to select the best fit "minimal" model for each pathogen, allowing candidate models to include a smoothed weekly time trend and up to two smoothed behavioral terms. Candidate predictors included between-neighborhood movement, inflow from other US states, the percentage of devices leaving home, and foot traffic to various categories of POIs, including restaurants, religious organizations, elementary and high schools, and colleges. For SARS-CoV-2, we also included the proportion of individuals masking in public and NPI stringency as candidate predictors. After model selection of candidate models, parameter estimation of the final model was performed by restricted maximum likelihood.

For each pathogen in each time period, the final GAM can be formally written as:

$$\log(\mathbb{E}(R_t)) = \beta_0 + f_1(Behavior1_t) + f_2(Behavior2_t) + f_3(week_t) + \varepsilon_t, \quad (14)$$

where R_t is the daily effective reproduction number, β_0 is the model intercept, f_j are smooth nonparametric functions of model covariates, and ε_t are identically and independently distributed (i.i.d.) model errors (residuals). For daily time series data, the assumption of independence of model residuals is often violated[18]. To account for temporal autocorrelation of residuals, we evaluated GAMs that included a continuous time first-order autoregressive process (CAR(1) with correlation parameter ϕ), with and without a smoothed weekly time trend. GAMs plus CAR(1) experienced convergence issues and produced poorer fits to the data, potentially because our models were fit to 2- to 6-month timespans, and fluctuations in the dependent variable and strong autocorrelation ($\phi \to 1$) can be unidentifiable when they occur on similar time scales[18].

Short-term forecasting of daily transmissibility

We built forecasting models predicting daily R_t at one-week horizons for three viruses that circulated continuously throughout the study period: hRV, AdV, and SARS-CoV-2. Candidate predictors included cellphone derived mobility metrics, the co-circulation of other viruses, and climatic variables, and past activity of the target virus during the previous two weeks (14 autoregressive terms). We evaluated an additional model for SARS-CoV-2 spanning 2021-2022 that included covariates for vaccination coverage and variant emergence (Fig. S22).

Similar to an approach for forecasting influenza-like illness activity (AutoRegression with GOogle search data)[19], our models implemented L1 regularization (LASSO) to automatically select the most relevant terms for predicting R_t up to 7-days ahead, using a moving window for the training period (that immediately precedes the dates of estimation) to capture the most recent changes in human mobility, weather, and viral activity[19]. For all three viruses, we found that one-month moving windows produced the most accurate forecasts of R_t, though there were few discernible trends in which mobility terms training windows retained over time. Expanding the training window

produced clearer patterns of which mobility terms were consistently retained by models but at the expense of predictive accuracy.

Model covariates. Candidate predictors included the activity of the target virus during the previous two weeks (14 autoregressive terms) and 1-, 7-, and 14-day lags of cellphone mobility metrics, masking rates, NPI stringency, climatic variables, and co-circulation of other viruses. For each virus, we included behavioral metrics that were associated with daily transmissibility in univariate cross-correlation analyses. For hRV and AdV models, behavioral predictors included between-neighborhood movement, visitor inflow from other WA counties and US states, the percentage of devices leaving home, and foot traffic to religious organizations, child day cares, schools, and colleges. For SARS-CoV-2 models, behavioral predictors included visitor inflow from other WA counties and US states, the percentage of devices leaving home, foot traffic to restaurants and religious organizations, the proportion of individuals masking in public, and the Oxford Stringency Index. Climatic variables included daily records of precipitation, average wet bulb temperature, and average relative humidity in Seattle, Washington (station ID: 72793024233), obtained from the National Centers for Environmental Information's U.S. Local Climatological Database[20]. To approximate virus-virus interactions, we included SARS-CoV-2 R_t as a covariate in the hRV and AdV models, and hRV R_t as a covariate in the SARS-CoV-2 models.

We evaluated an additional model predicting SARS-CoV-2 R_t for dates spanning 2021-2022 that included covariates for cumulative COVID-19 vaccination coverage and variant circulation in King County, WA. We estimated daily cumulative vaccination coverage as the cumulative proportion of eligible King County residents (people at least 5 years of age) who were fully vaccinated for COVID-19, defined as having received one Johnson & Johnson (Janssen) vaccine dose or at least two mRNA vaccine doses. COVID-19 vaccination data were obtained from the Public Health – Seattle & King County COVID-19 Vaccination dashboard[21].

To estimate daily SARS-CoV-2 variant frequencies, we downloaded Nextstrain-curated SARS-CoV-2 sequence metadata[22], which is created using the GISAID EpiCoV database[23]. We filtered the dataset to sequences collected in King County, WA during February 2020 to June 2022 with "good" quality control (QC) scores (N = 9,626 genomes). We used multinomial logistic regression to model the daily frequencies of SARS-CoV-2 clades circulating in King County, including the ancestral virus and major variants of concern (VOCs), based on the observed frequencies of Nextstrain clades in the dataset and the number of days since the first detection (Fig. S22).

First, we drew the number of clade-specific genomes from a multinomial distribution:

$$Y_{c,t} \sim \text{Multinomial}(S_t, \pi_{c,t}), \tag{15}$$

where $Y_{c,t}$ is the number of sequences on day *t* belonging to clade *c*, S_t is the total number of sequences collected on day *t*, and $\pi_{c,t}$ is the probability of a sequence on day *t* belonging to clade *c*.

$\pi_{c,t}$ is equal to the observed frequency of clade *c* on day *t*:

$$\pi_{c,t} = \frac{X_{c,t}}{S_t} \tag{16}$$

We estimated the sample probability of clade *c* on day *t* as the predicted number of sequences belonging to clade *c* divided by the total number of sequences collected on day *t*:

$$P_{c,t} = \frac{Y_{c,t}}{S_t} \tag{17}$$

To capture the effects of variant emergence on R_t, the 2021-2022 model included the daily predicted frequencies of sequences belonging to the Alpha variant, Delta variant, or Omicron BA.1 variant.

Model evaluation. For each virus, we evaluated the predictive power of cellphone derived mobility metrics, the co-circulation of other viruses, and climatic variables, in combination with past activity of the target virus during the previous two weeks (14 autoregressive terms), against a baseline model that included only the past activity of the

target virus (14 AR terms). For each model at each forecast date, we calculated the percentage error as the difference between the predicted and observed values of R_t, divided by the observed value of R_t. Positive values of percentage error indicate overprediction, and negative values indicate underprediction. We evaluated overall model performance by comparing the root-mean-squared-error (RMSE) and mean absolute error (MAE) scores of model predictions against the observed R_t values (Tables S3-S5). We estimated the benefit of including variables related to mobility, climate, or viral interactions by calculating the percent difference in RMSE and MAE relative to the baseline model, wherein negative values indicate models with additional covariates are more accurate, and positive values indicate the baseline model is more accurate (Tables S3-S5). Model performance across the whole study period is reported in Table S3, model performance during Seattle's COVID-19 stay-at-home orders and the initial lifting of restrictions is reported in Table S4, and model performance for SARS-CoV-2 R_t during 2021-2022 is reported in Table S5.

Supplementary Results

Short-term forecasts of rhinovirus (hRV), adenovirus (AdV), and SARS-CoV-2 transmission

For all three viruses, models including mobility and AR terms produced generally accurate forecasts over the entire study period (RMSE: hRV: 0.013; AdV: 0.04; SARS-CoV-2: 0.03; Table S3), and especially during Seattle's stay-at-home (SAH) orders and the initial lifting of restrictions (RMSE: hRV: 0.006; AdV: 0.02; SARS-CoV-2: 0.02; Table S4). For models predicting SARS-CoV-2 R_t during 2021-2022, including covariates for vaccination and variant emergence did not improve prediction accuracy, but models with mobility had a 13% improvement in prediction RMSE relative to the baseline model (Table S5). Among candidate mobility predictors, the percentage of devices leaving home, between-neighborhood movement, and visitor inflow had the highest mean (absolute) coefficient values and were the most frequently retained variables across moving training windows. In models predicting SARS-CoV-2 R_t, masking and NPI stringency were also frequently retained and had high mean coefficient values.

When comparing model accuracy over the entire study period, models including covariates for mobility, climate, or viral interference did not outperform baseline models for hRV and AdV (Figs. S19-S21; Table S3). For SARS-CoV-2, the model including prior information on precipitation, temperature, and relative humidity performed similarly to the baseline model (< 1% difference in prediction RMSE; Table S3) yet moving training windows did not frequently retain climatic variables (< 110 out of 758 windows), and climatic variables had the smallest model coefficient values among candidate predictors (range: 0.0000013 – 0.00017, compared to 0.002 – 0.003 for the Oxford Stringency Index). Thus, we did not find definitive evidence that meteorological information improves forecasts of R_t for any virus, potentially because non-enveloped viruses and pandemic SARS-CoV-2 do not exhibit strong seasonality. Although models with mobility and AR terms were 46% (AdV), 52% (hRV), and 26% (SARS-CoV-2) more accurate during Seattle's SAH orders compared to the entire study period, models for hRV and AdV still underperformed in comparison to baseline models during these months (Table S3).

Overall, we found that prior disease activity alone is most beneficial for accurately projecting future transmission dynamics. Tracking mobility behavior is not essential for forecasting respiratory virus transmission, and the inclusion of mobility data can even be detrimental to prediction accuracy, depending on the pathogen and time period (Tables S3-S5). Monitoring major changes in mobility could still be helpful for general situational awareness and planning purposes in the early stages of an emerging disease outbreak, when testing capacity is low and the true incidence of the disease is unknown. However, prior information on mobility trends is unlikely to provide a net benefit to prediction accuracy when an epidemic is widely established in a population. This finding is consistent with another study that used a different modeling approach and set of mobility metrics to forecast COVID-19 cases and deaths in Europe[24].

Supplementary Figures

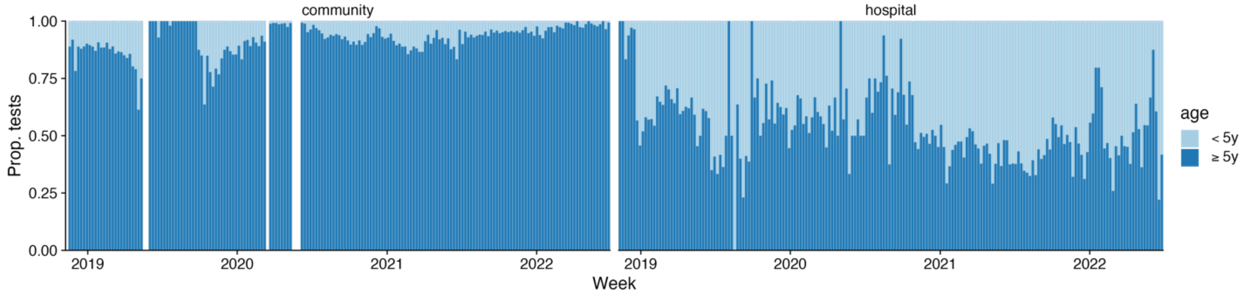

Fig. S1. The weekly age distribution of respiratory specimens collected from community and hospital settings, November 2018 – June 2022. Bar colors show the proportion of samples collected from individuals aged <5 (light blue) or ≥5 years (dark blue). Sample sources for community-based testing include swab-and-send at-home testing programs, kiosks in high foot traffic areas, outpatient clinics, and Public Health – Seattle & King County COVID-19 drive-through testing sites.

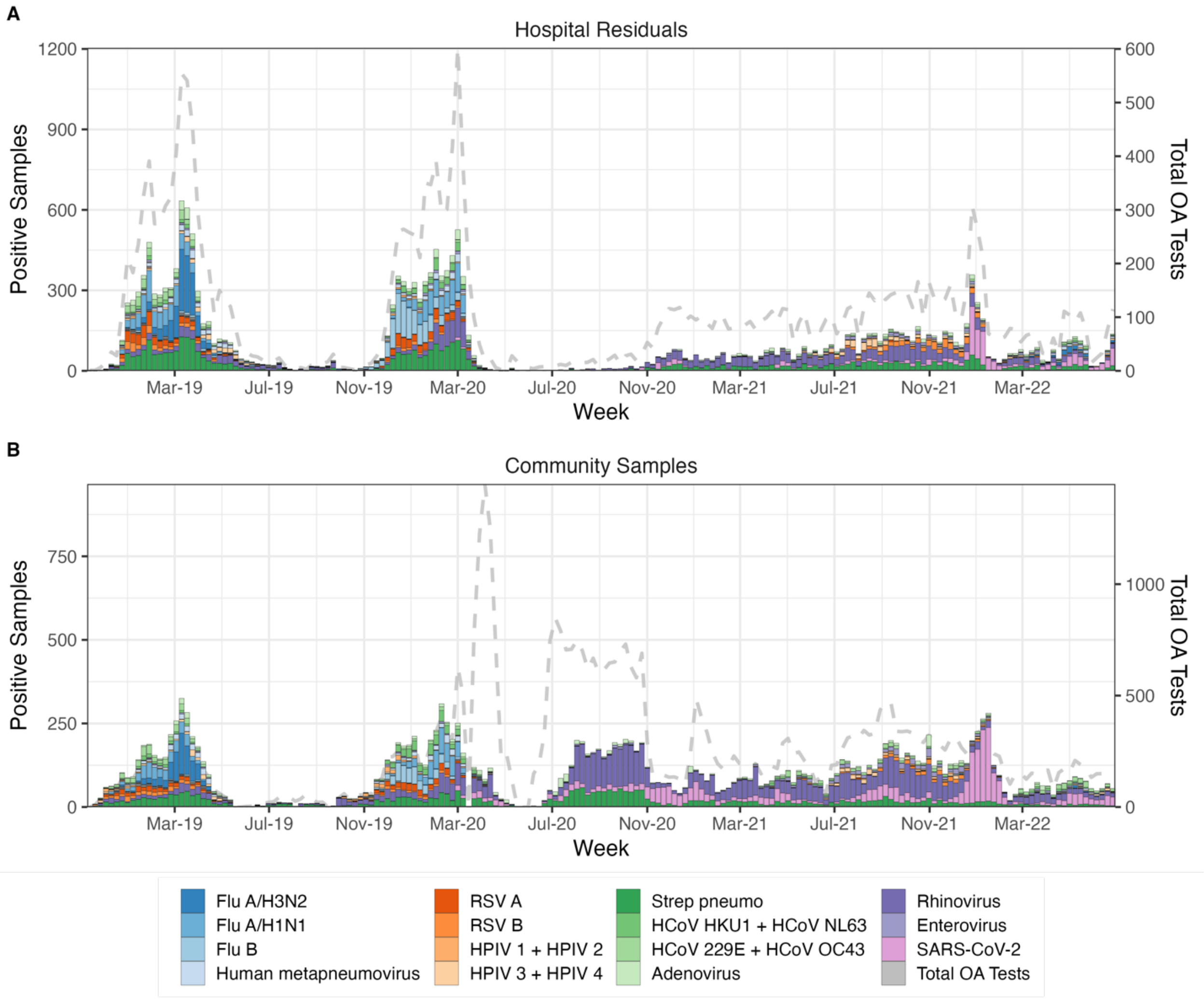

Fig. S2. The weekly number of samples testing positive for respiratory pathogens in A. hospitals and B. community settings. Colored bars represent the number of samples testing positive for each pathogen. The gray dashed line is the number of respiratory specimens tested on the OpenArray (OA) platform. The left y-axis corresponds to the number of positive samples collected each week, and the right y-axis corresponds to the total number of specimens collected each week and tested on OA. Sources for community-based samples include swab-and-send at-home testing programs, kiosks in high foot traffic areas, outpatient clinics, and Public Health – Seattle & King County COVID-19 drive-through testing sites.

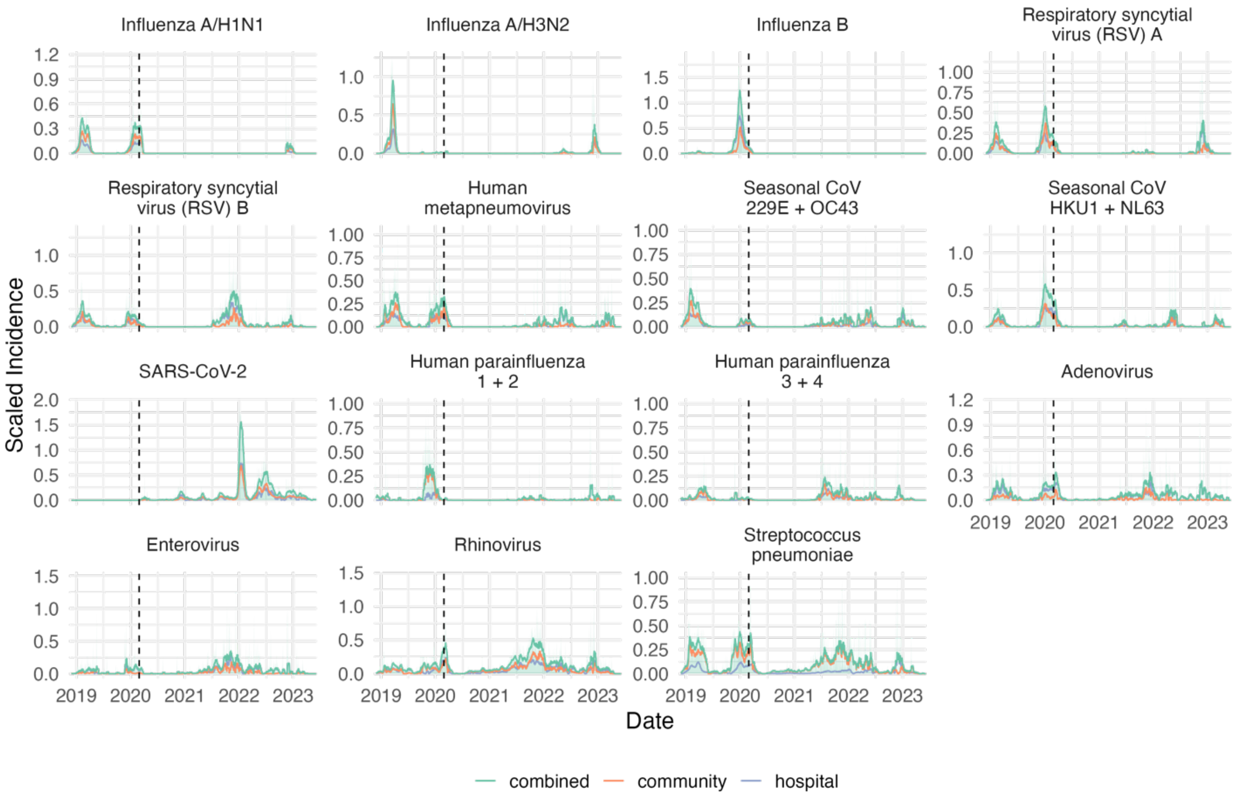

Fig. S3. Reconstructed daily incidences of individual respiratory pathogens, adjusted for testing volume over time, age, clinical setting, and local syndromic respiratory illness rates. Community and hospital-based incidences (orange and purple lines, respectively) were rescaled to fall between 0 and 1 and summed (green lines) to aid in comparing relative changes in incidence between pathogens over time. To reduce noise, we applied centered two-week moving averages to incidences. The vertical dashed line indicates the date of Washington's State of Emergency declaration (February 29, 2020).

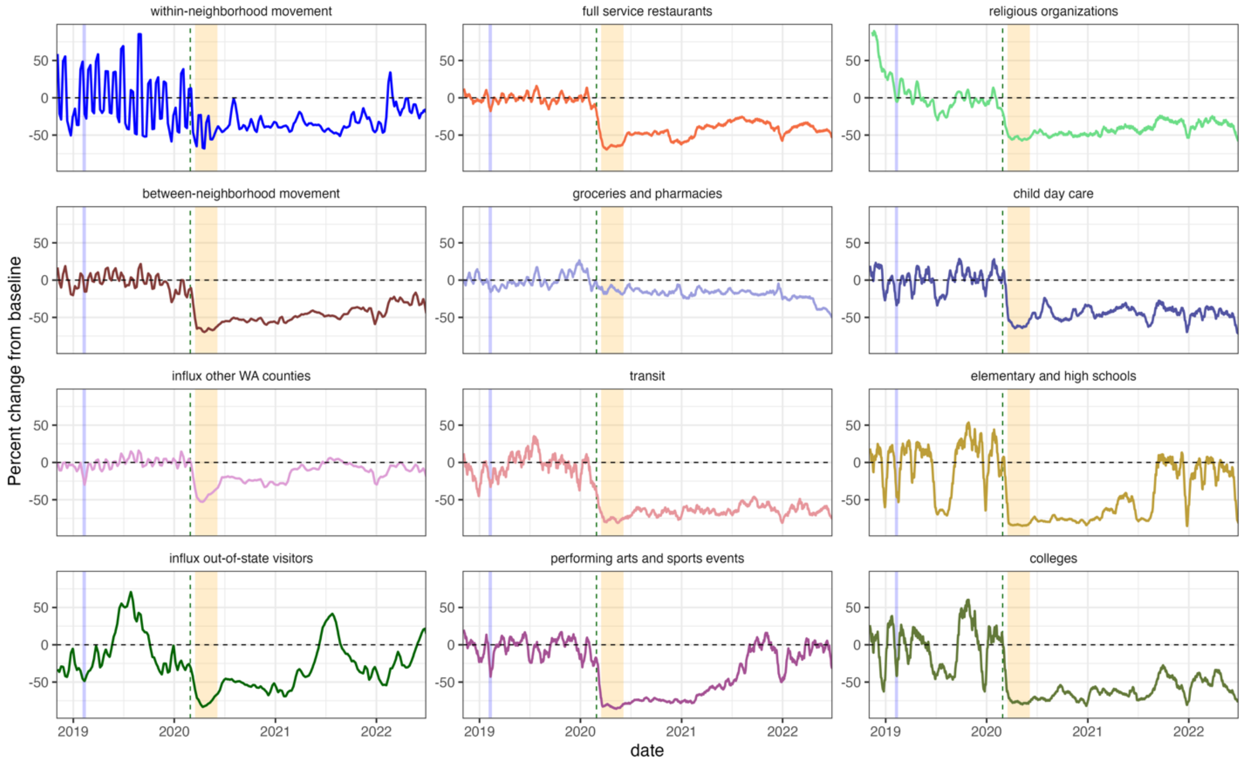

Fig. S4. Mobility metrics derived from SafeGraph mobile device location data for the greater Seattle region, November 2018 – June 2022. For each mobility indicator, we summed daily or weekly visits for each point of interest (POI) category and measured the percent change in movement over time relative to the average movement observed in all of 2019 (excluding national holidays). We applied a centered two-week moving average to each metric to reduce noise. In each facet, the vertical blue shaded panel indicates the timing of a major snowstorm in Seattle (February 3-15, 2019), the vertical dashed line indicates the date of Washington's State of Emergency declaration (February 29, 2020), and the vertical orange shaded panel indicates Seattle's stay-at-home period (March 23 – June 5, 2020).

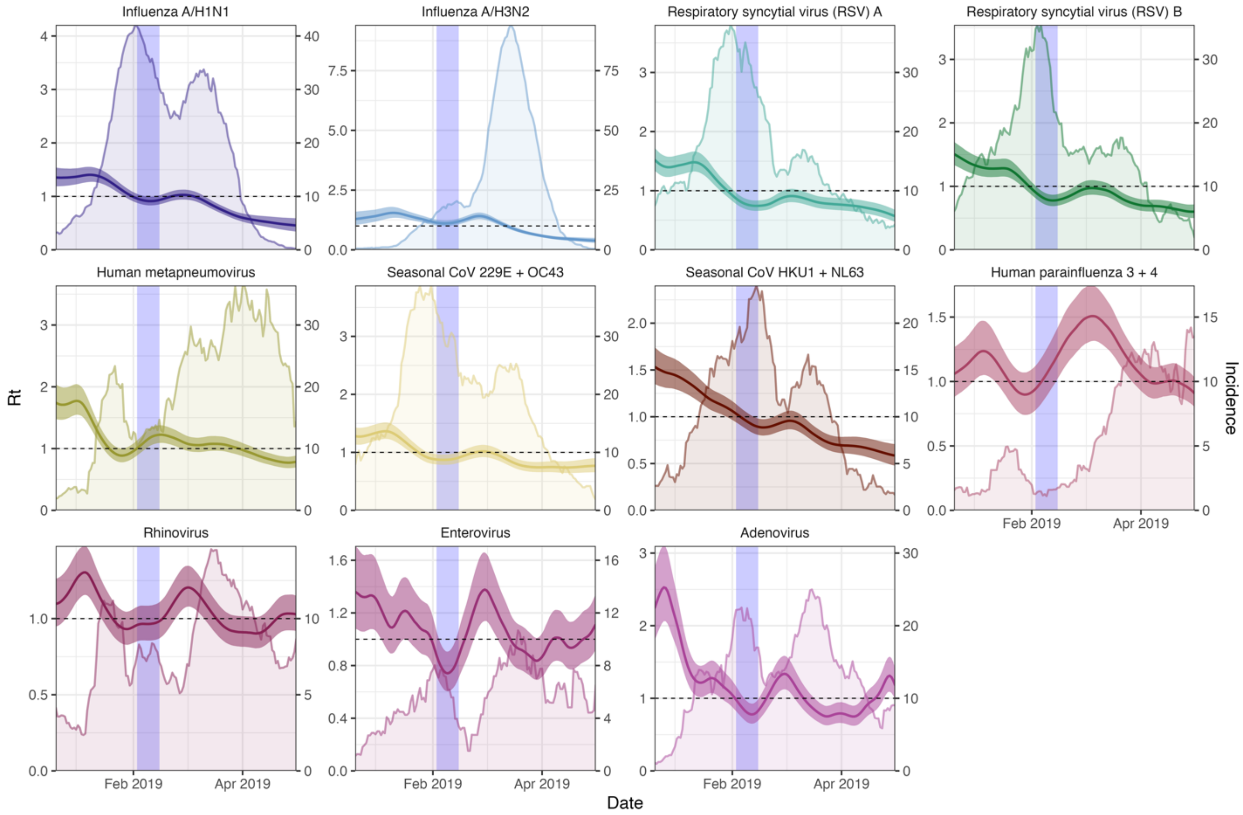

Fig. S5. Daily incidences and transmissibility of respiratory viruses circulating in the greater Seattle region, December 2018 – May 2019. Daily time-varying effective reproduction numbers (R_t, thick lines, left y-axis) and reconstructed incidences of respiratory viruses (thin lines, right y-axis). Daily R_t time series show the posterior median (thin dark line) and 90% credible interval (shaded band). We applied centered two-week moving averages to daily incidences to reduce noise. The vertical blue shaded panel indicates the timing of a major snowstorm (February 3 – 15, 2019).

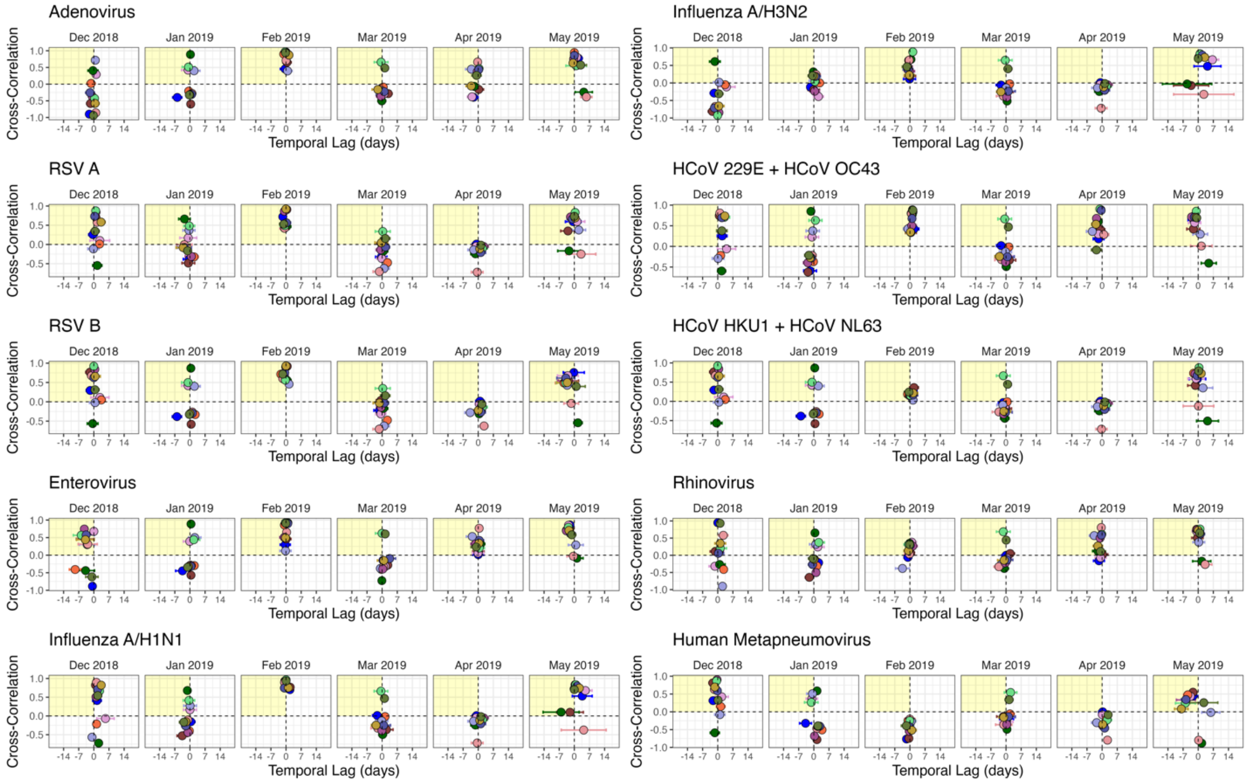

Fig. S6. Time series cross-correlations and optimal lags between respiratory pathogen transmissibility (daily effective reproduction numbers, R_t) and cell phone mobility in the greater Seattle region, December 2018 – May 2019. Points are individual mobility indicators derived from SafeGraph mobile device location data. Daily cross correlations in rolling 1-month windows were averaged by calendar month. Horizontal error bars are the 95% confidence intervals of optimal lags within each calendar month. Spearman correlation coefficients are on the y-axis, and temporal lags (in days) between R_t and mobility are on the x-axis. Negative temporal lags indicate behavior leads R_t, and positive temporal lags indicate R_t leads behavior. A lag of 0 indicates the time series are in phase (i.e., synchronous). The yellow shaded panel in each facet includes mobility indicators that have a leading, positive relationship with transmission, and hence would be considered predictive of transmission.

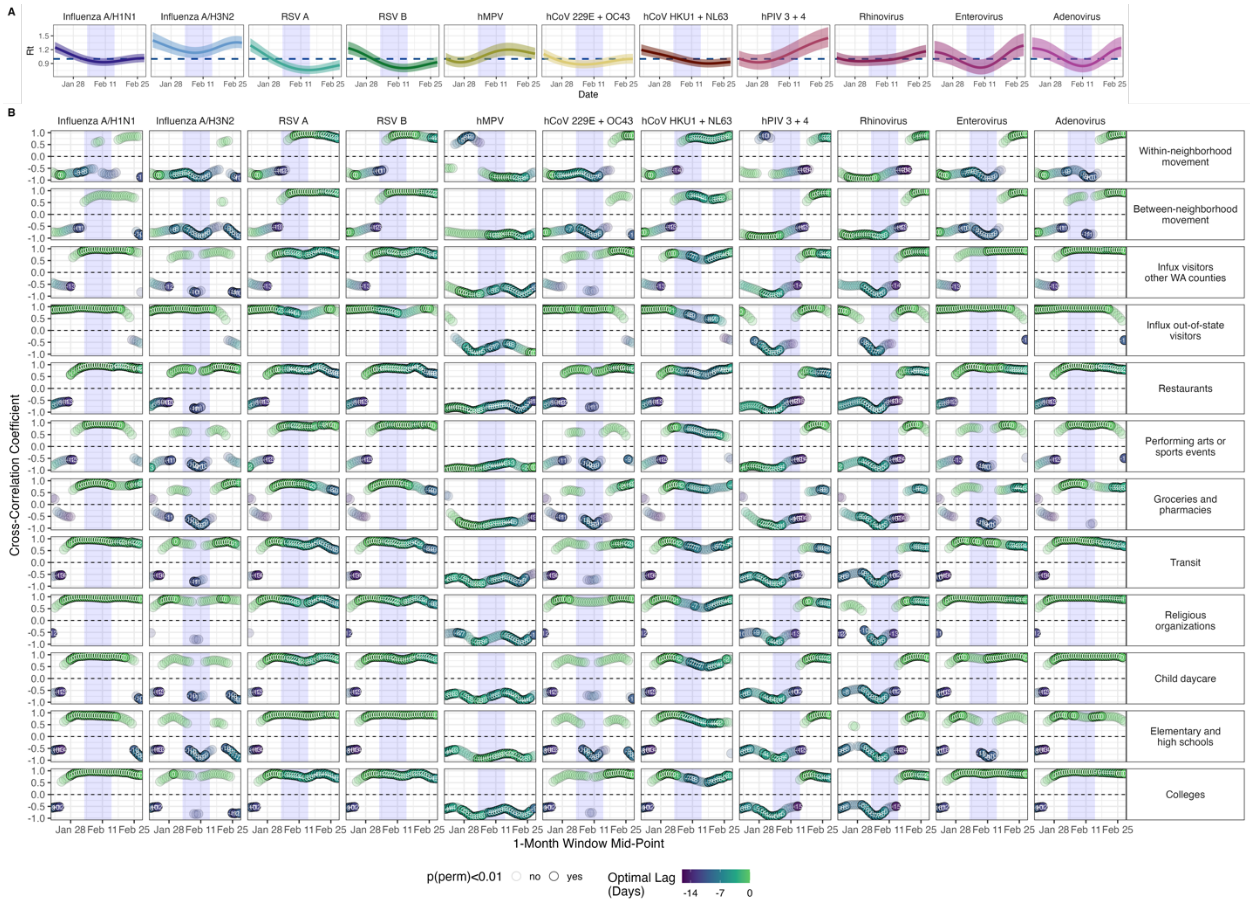

Fig. S7. A. Daily effective reproduction numbers (R_t) of respiratory viruses circulating in the greater Seattle region, and B. Rolling daily cross-correlations between pathogen transmissibility and cell phone mobility during January – February 2019. Daily R_t time series show the posterior median (thin dark line) and 90% credible interval (shaded band). Points represent the maximum (absolute) coefficient values for 1-month rolling Spearman cross-correlations between daily effective reproduction numbers (R_t) and individual mobility metrics, when constraining the analysis to leading or synchronous relationships between mobility and R_t. Point color and the number within each point indicate the lag in days corresponding to the maximum cross-correlation coefficient value for each 1-month window ("optimal lag"). Negative values indicate that mobility leads R_t, and a lag of 0 indicates the time series are in phase (i.e., synchronous). Point transparency indicates statistical significance based on 1000 block bootstrap permutations (yes: solid, no: transparent). The vertical blue shaded panel indicates the timing of a major snowstorm (February 3 – 15, 2019).

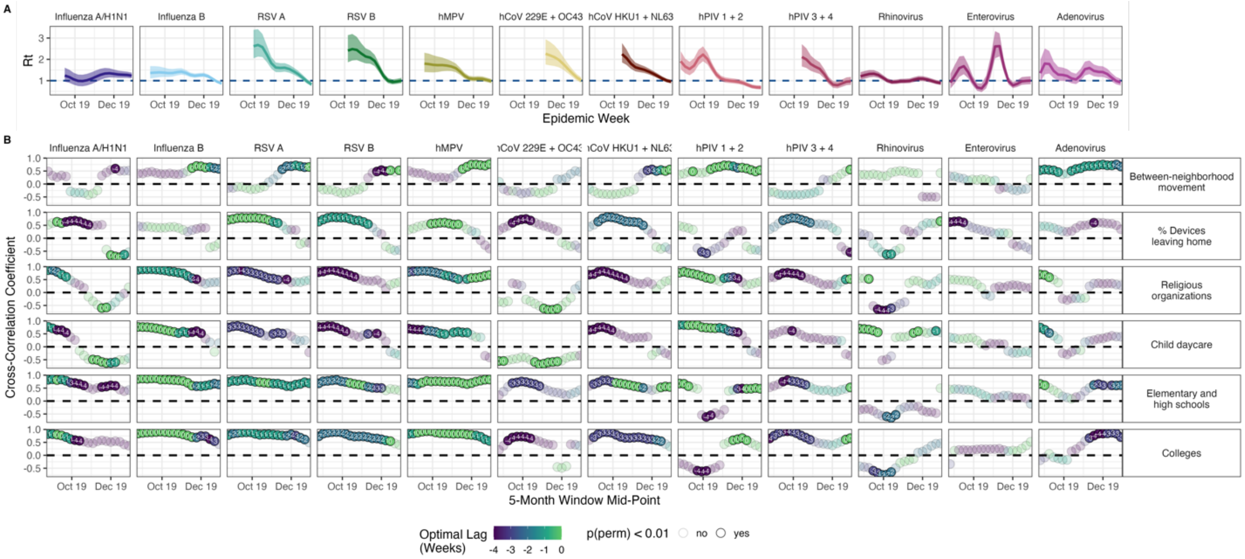

Fig. S8. A. Weekly effective reproduction numbers (R_t) of respiratory viruses circulating in the greater Seattle region, and B. Rolling cross-correlations between pathogen transmissibility and cell phone mobility during the 2019-2020 winter season prior to the start of the COVID-19 pandemic, August 2019 – December 2020. Weekly R_t time series show the posterior median (thin dark line) and 90% credible interval (shaded band). Points represent the maximum (absolute) coefficient values for 5-month rolling Spearman cross-correlations between weekly effective reproduction numbers (R_t) and individual mobility metrics, after constraining the analysis to leading or synchronous relationships between mobility and R_t. Point color and the number within each point indicate the lag in weeks corresponding to the maximum cross-correlation coefficient value for each 5-month period (“optimal lag”). Negative values indicate that mobility leads R_t, and a lag of 0 indicates the time series are in phase (i.e., synchronous). Point transparency indicates statistical significance based on 1000 block bootstrap permutations (yes: solid, no: transparent).

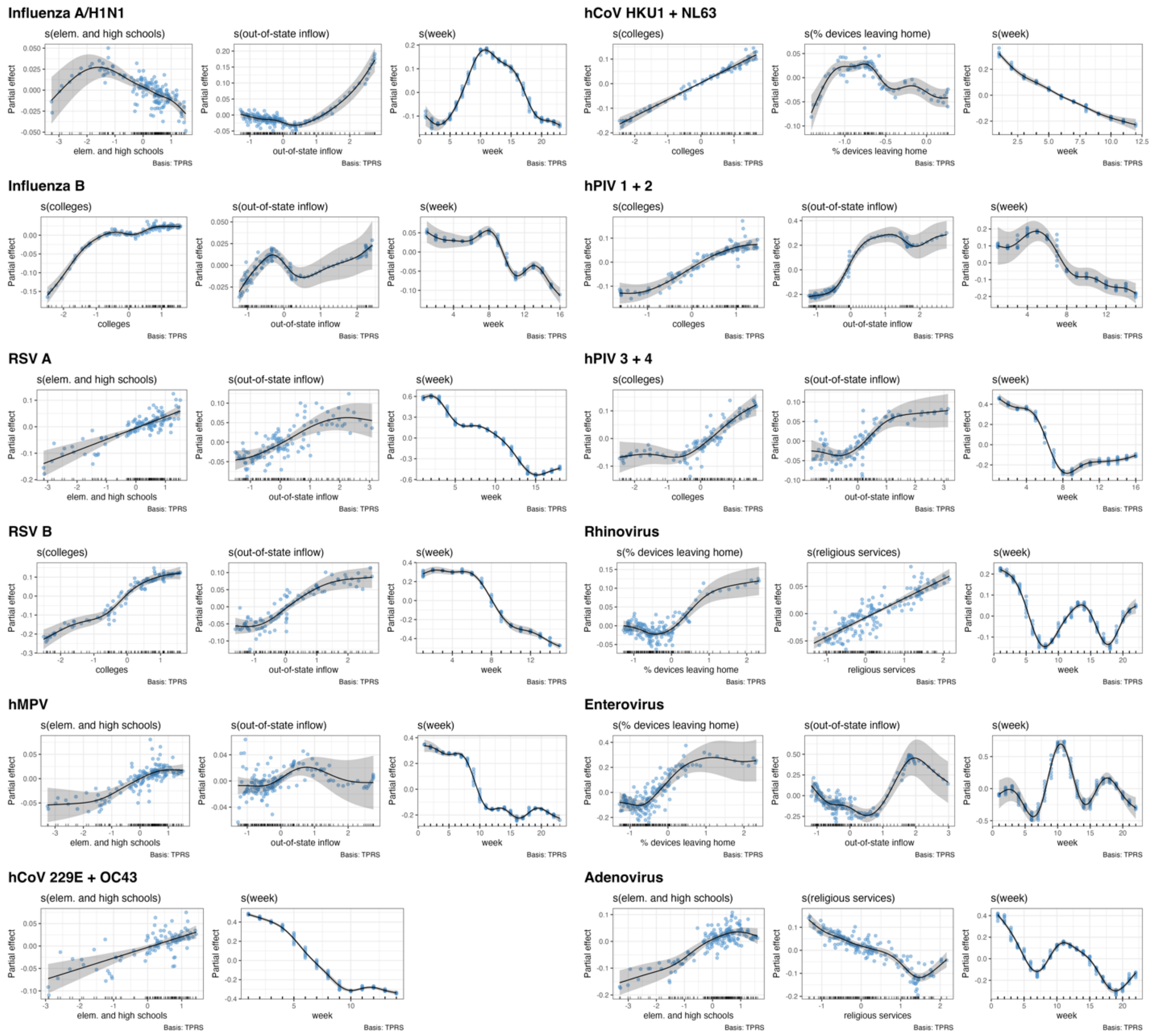

Fig. S9. Generalized additive model (GAM) plots showing the partial effects of selected mobility indicators and time trends on the daily effective reproduction numbers (R_t) of endemic respiratory viruses during the 2019-2020 winter season prior to the start of the COVID-19 pandemic, September 2019 – February 2020. GAMs were fit to the exponential growth phase of each wave, when R_t exceeds 1. Tick marks on the x-axis indicate observed data points. The y-axis represents of the partial effect of each variable. Gray shaded bands are the 95% confidence intervals of partial effects. The blue points are partial residuals.

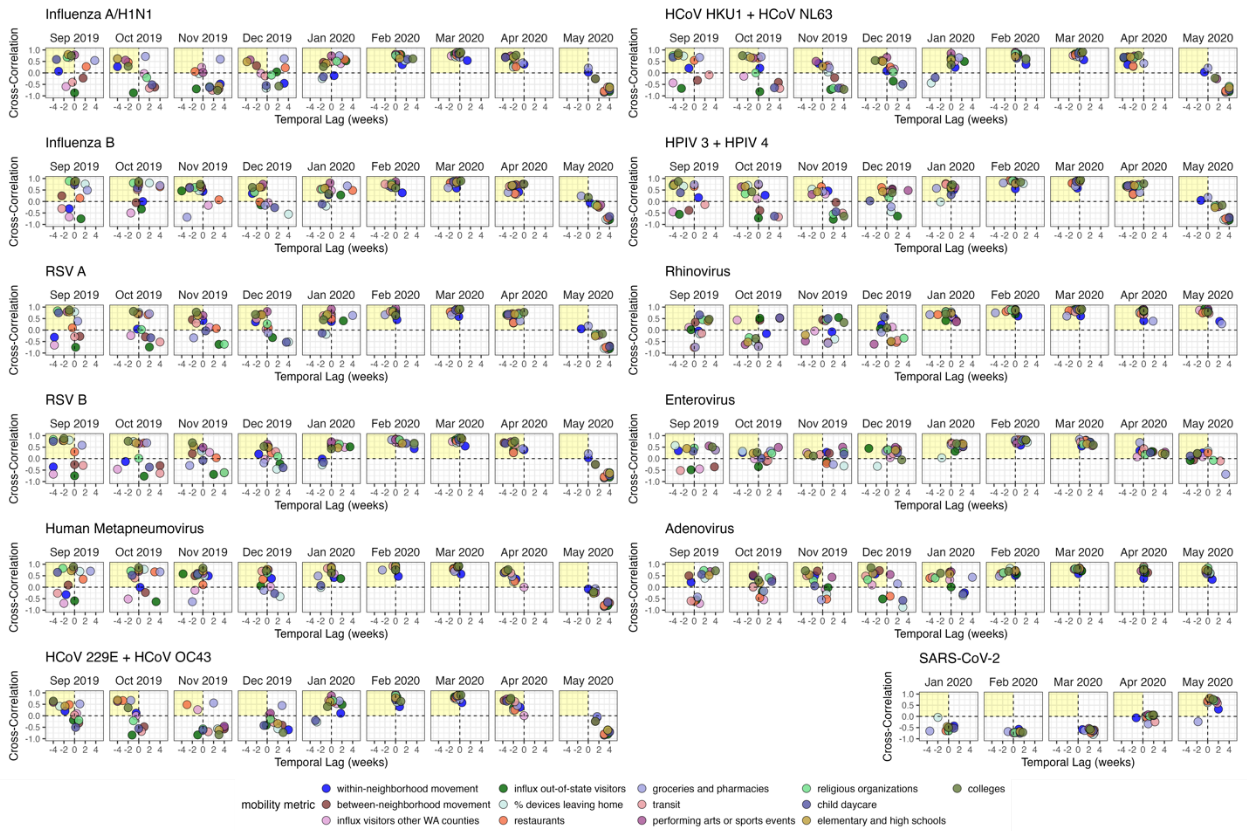

Fig. S10. Time series cross-correlations and optimal lags between respiratory virus transmissibility (time-varying effective reproduction numbers, R_t) and cell phone mobility in the greater Seattle region, September 2019 – May 2020. Points are individual mobility indicators derived from aggregated mobile device location data. Spearman correlation coefficients are on the y-axis, and temporal lags (in weeks) between R_t and mobility are on the x-axis. Negative lags indicate behavior leads R_t, positive lags indicate R_t leads behavior, and a lag of 0 indicates the two time series are in phase (i.e., synchronous). The yellow shaded panel in each facet includes mobility indicators that have a leading, positive relationship with transmission, and hence would be considered predictive of transmission.

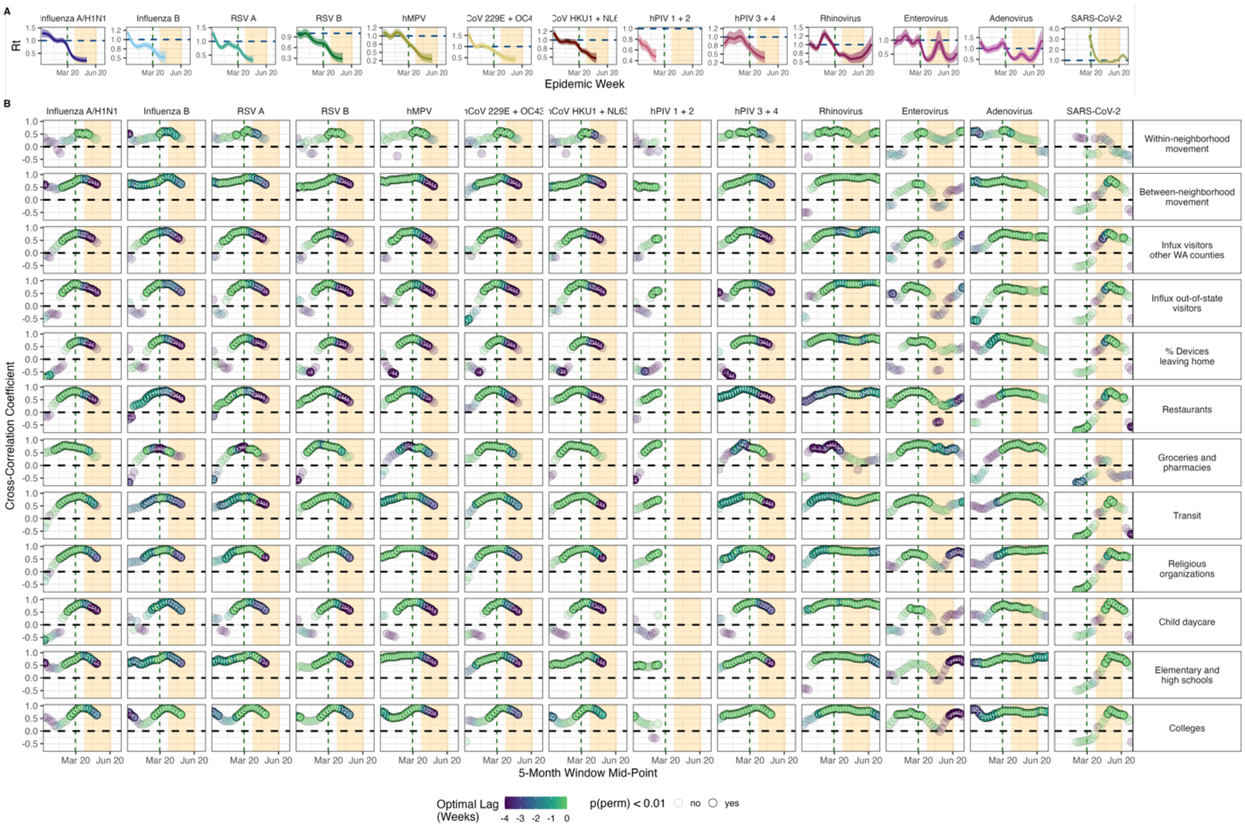

Fig. S11. A. Weekly effective reproduction numbers (R_t) of respiratory viruses circulating in the greater Seattle region, and B. Rolling cross-correlations between pathogen transmissibility and cell phone mobility during the early months of the COVID-19 pandemic, December 2019 – June 2020. Weekly R_t time series show the posterior median (thin dark line) and 90% credible interval (shaded band). Points represent the maximum (absolute) coefficient values for 5-month rolling Spearman cross-correlations between weekly effective reproduction numbers (R_t) and individual mobility metrics, after constraining the analysis to leading or synchronous relationships between mobility and R_t. Point color and the number within each point indicate the lag in weeks corresponding to the maximum cross-correlation coefficient value for each 5-month period ("optimal lag"). Negative values indicate that mobility leads R_t, and a lag of 0 indicates that the time series are in phase. Point transparency indicates statistical significance based on 1000 block bootstrap permutations (yes: solid, no: transparent). The vertical dashed line indicates the date of Washington's State of Emergency declaration (February 29, 2020), and the vertical orange shaded panel indicates Seattle's stay-at-home period (March 23 – June 5, 2020).

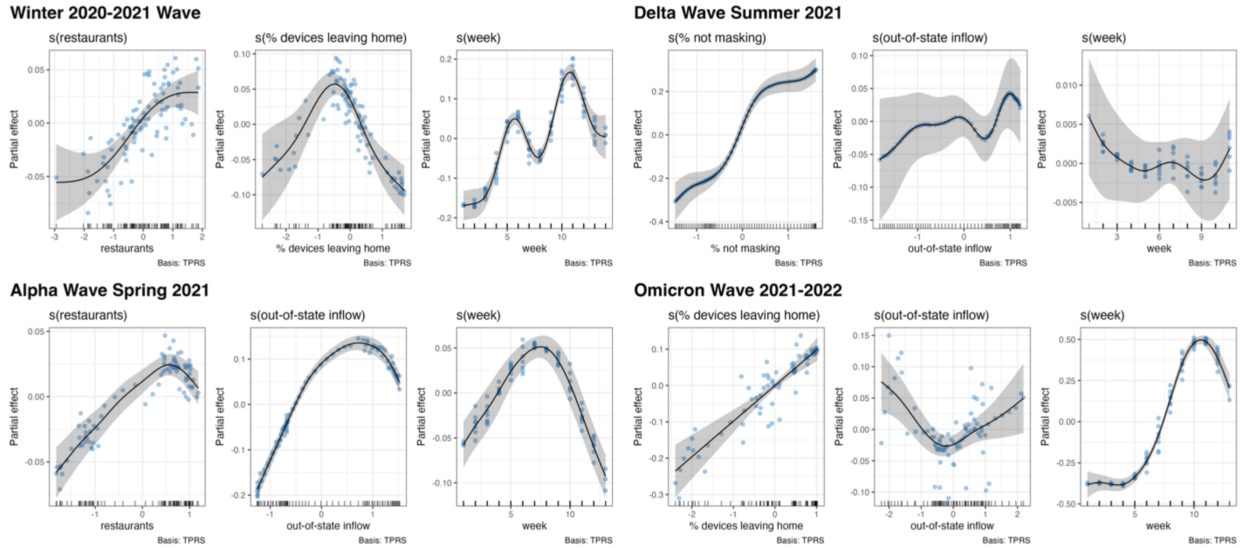

Fig. S12. Generalized additive model (GAM) plots showing the partial effects of selected mobility indicators and time trends on the daily effective reproduction numbers (R_t) of SARS-CoV-2 during four COVID-19 waves in Seattle: the winter 2020-2021 wave, the Alpha wave in Spring 2021, the Delta wave in Summer 2021, and the Omicron BA.1 wave during late 2021 to early 2022. GAMs were fit to the exponential growth phase of each wave, when R_t exceeds 1. Tick marks on the x-axis indicate observed data points. The y-axis represents of the partial effect of each variable. Gray shaded bands are the 95% confidence intervals of partial effects. The blue points are partial residuals.

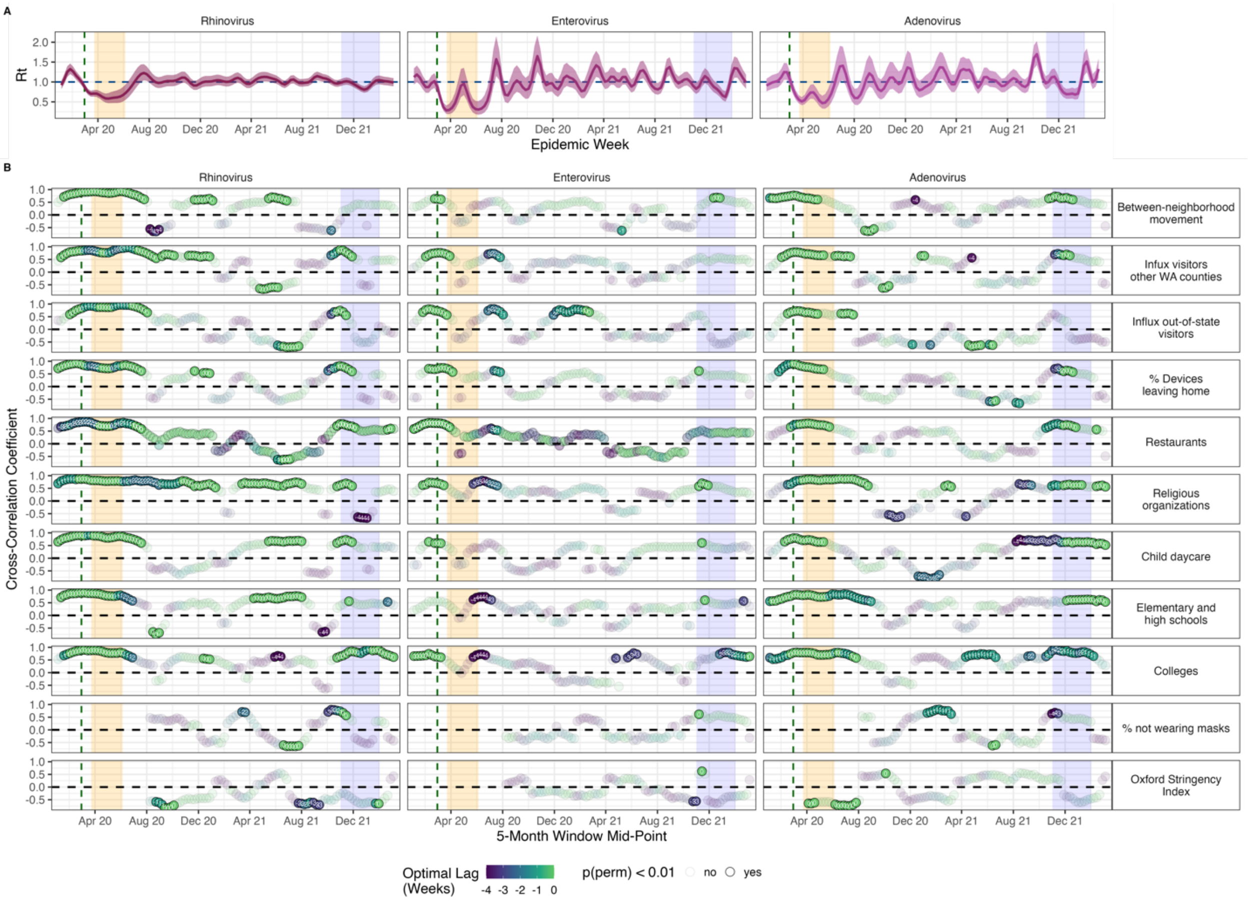

Fig. S13. A. Weekly effective reproduction numbers (R_t) of three non-enveloped viruses – rhinovirus, enterovirus, and adenovirus – circulating in the greater Seattle region, and B. Rolling cross-correlations between pathogen transmissibility and cell phone mobility during the COVID-19 pandemic, January 2020 – March 2022. Weekly R_t time series show the posterior median (thin dark line) and 90% credible interval (shaded band). Points are the maximum (absolute) coefficient values for 5-month rolling Spearman cross-correlations between weekly effective reproduction numbers (R_t) and individual behavioral metrics, after constraining the analysis to leading or synchronous relationships between mobility and R_t. Point color and the number within each point indicate the lag in weeks corresponding to the maximum cross-correlation coefficient value for each 5-month period (“optimal lag”). Negative values indicate that behavior leads R_t, and a lag of 0 indicates that the time series are in phase (i.e., synchronous). Point transparency indicates statistical significance based on 1000 block bootstrap permutations (yes: solid, no: transparent). The vertical dashed line indicates the date of Washington’s State of Emergency declaration (February 29, 2020), the vertical orange shaded panel indicates Seattle’s stay-at-home period (March 23 – June 5, 2020), and the vertical blue shaded panel indicates the timing of the Omicron BA.1 wave (November 2021 – January 2022).

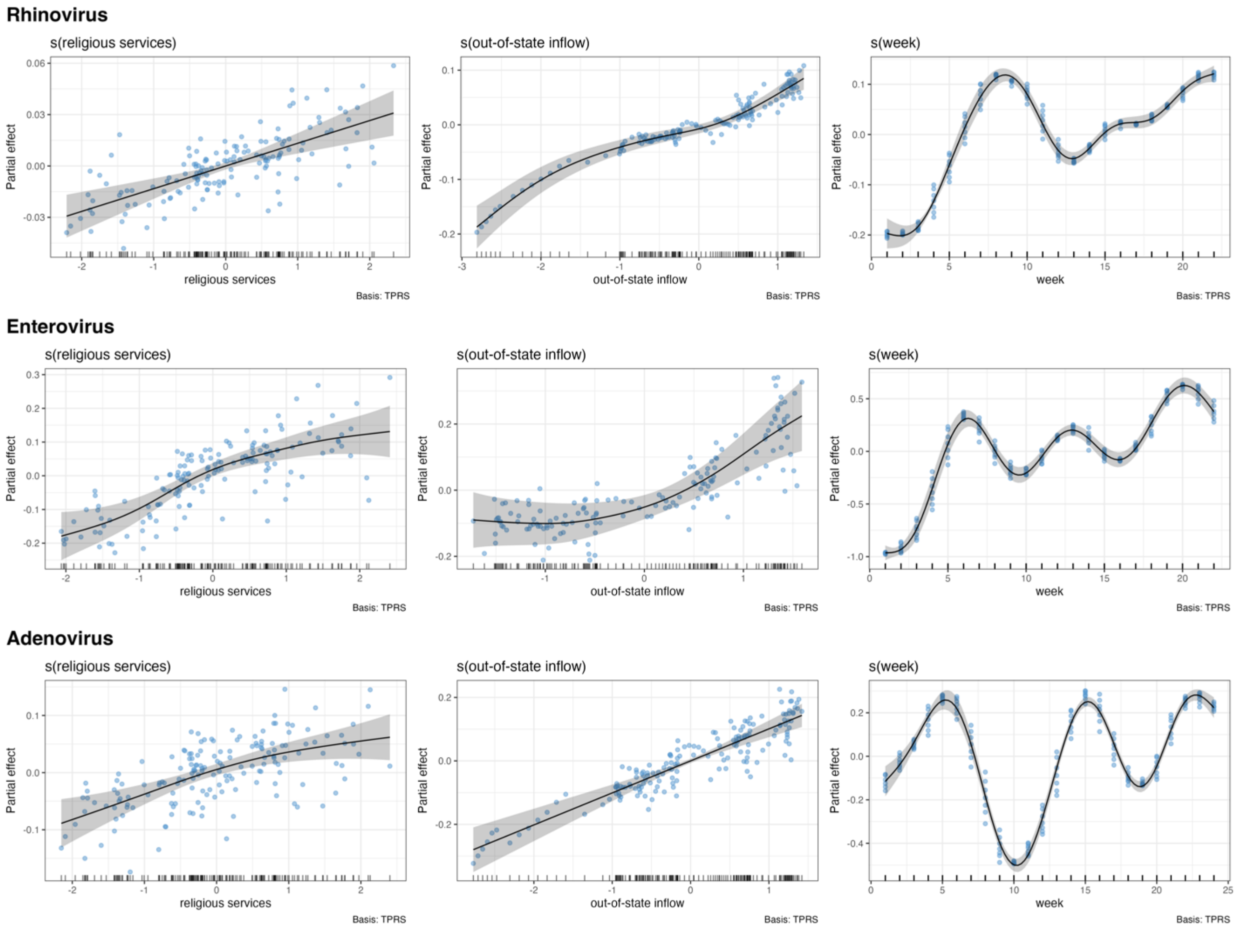

Fig. S14. Generalized additive model (GAM) plots showing the partial effects of selected mobility indicators and time trends on the daily effective reproduction numbers (R_t) of three non-enveloped respiratory viruses – rhinovirus, enterovirus, and adenovirus – during their first six months of rebound, June 2020 – November 2020. GAMs were fit to the exponential growth phase of each wave, when R_t exceeds 1. Tick marks on the x-axis indicate observed data points. The y-axis represents of the partial effect of each variable. Gray shaded bands are the 95% confidence intervals of partial effects. The blue points are partial residuals.

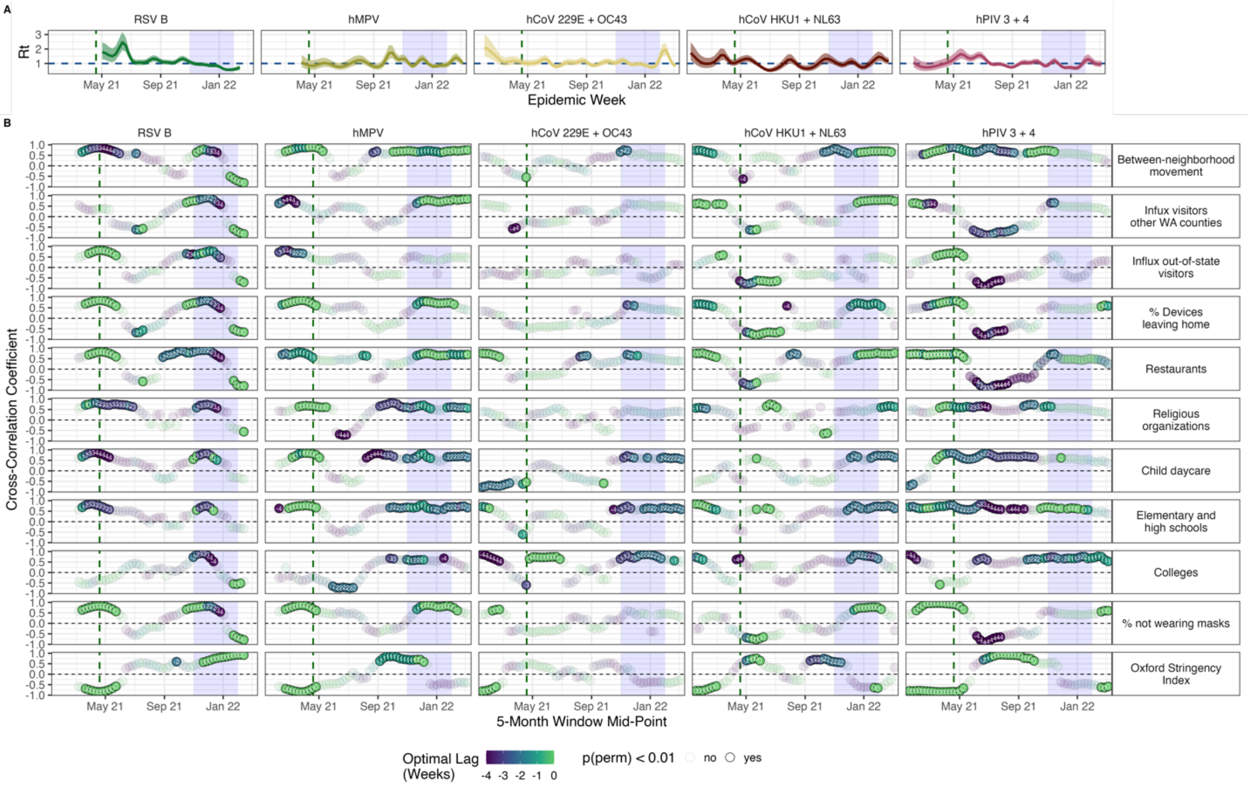

Fig. S15. A. Weekly effective reproduction numbers (R_t) of enveloped viruses circulating in the greater Seattle region, and B. Rolling cross-correlations between pathogen transmissibility and cell phone mobility during the COVID-19 pandemic, January 2021 – March 2022. Weekly R_t time series show the posterior median (thin dark line) and 90% credible interval (shaded band). Points are the maximum (absolute) coefficient values for 5-month rolling Spearman cross-correlations between weekly effective reproduction numbers (R_t) and individual mobility and behavioral metrics, after constraining the analysis to leading or synchronous relationships between behavior and R_t. Point color and the number within each point indicate the lag in weeks corresponding to the maximum cross-correlation coefficient value for each 5-month period ("optimal lag"). Negative values indicate that behavior leads R_t, and a lag of 0 indicates that the time series are in phase (i.e., synchronous). Point transparency indicates statistical significance based on 1000 block bootstrap permutations (yes: solid, no: transparent). The vertical dashed line indicates when Washington state required public schools to offer at least two days of partial in-person instruction to all grades (April 19, 2021), and the vertical blue shaded panel indicates the timing of the Omicron BA.1 wave (November 2021 – January 2022).

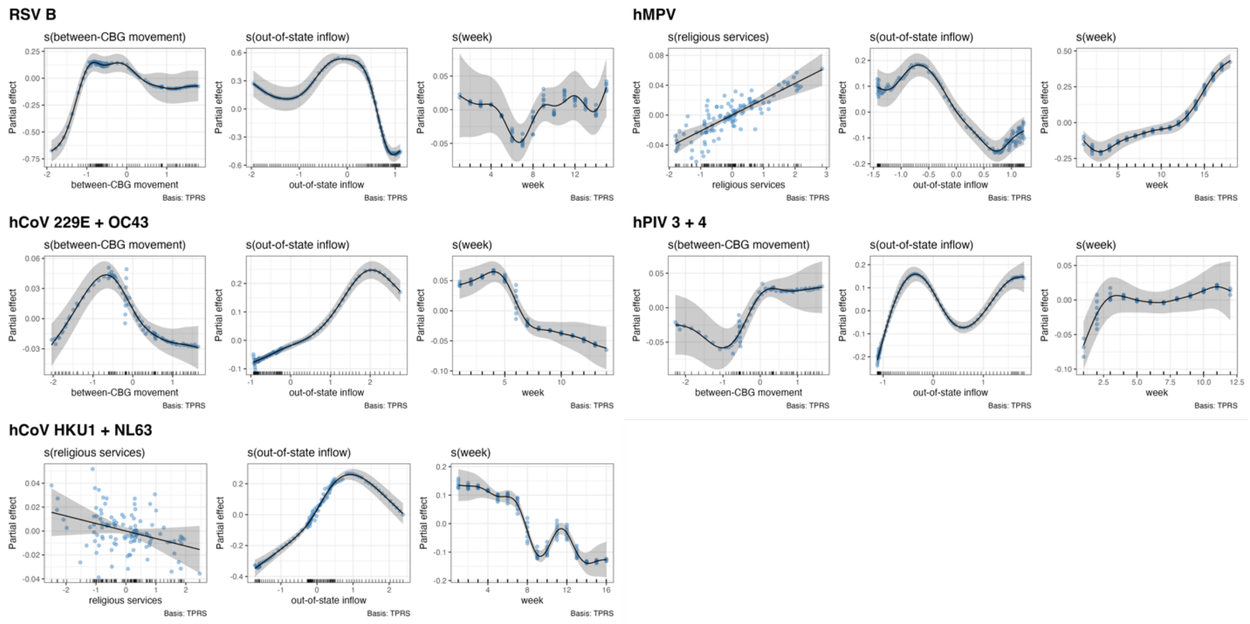

Fig. S16. Generalized additive model (GAM) plots showing the partial effects of selected mobility indicators and time trends on the daily effective reproduction numbers (R_t) of enveloped respiratory viruses during their initial months of rebound, January – August 2021. GAMs were fit to the exponential growth phase of each wave, when R_t exceeds 1. Tick marks on the x-axis indicate observed data points. The y-axis represents of the partial effect of each variable. Gray shaded bands are the 95% confidence intervals of partial effects. The blue points are partial residuals.

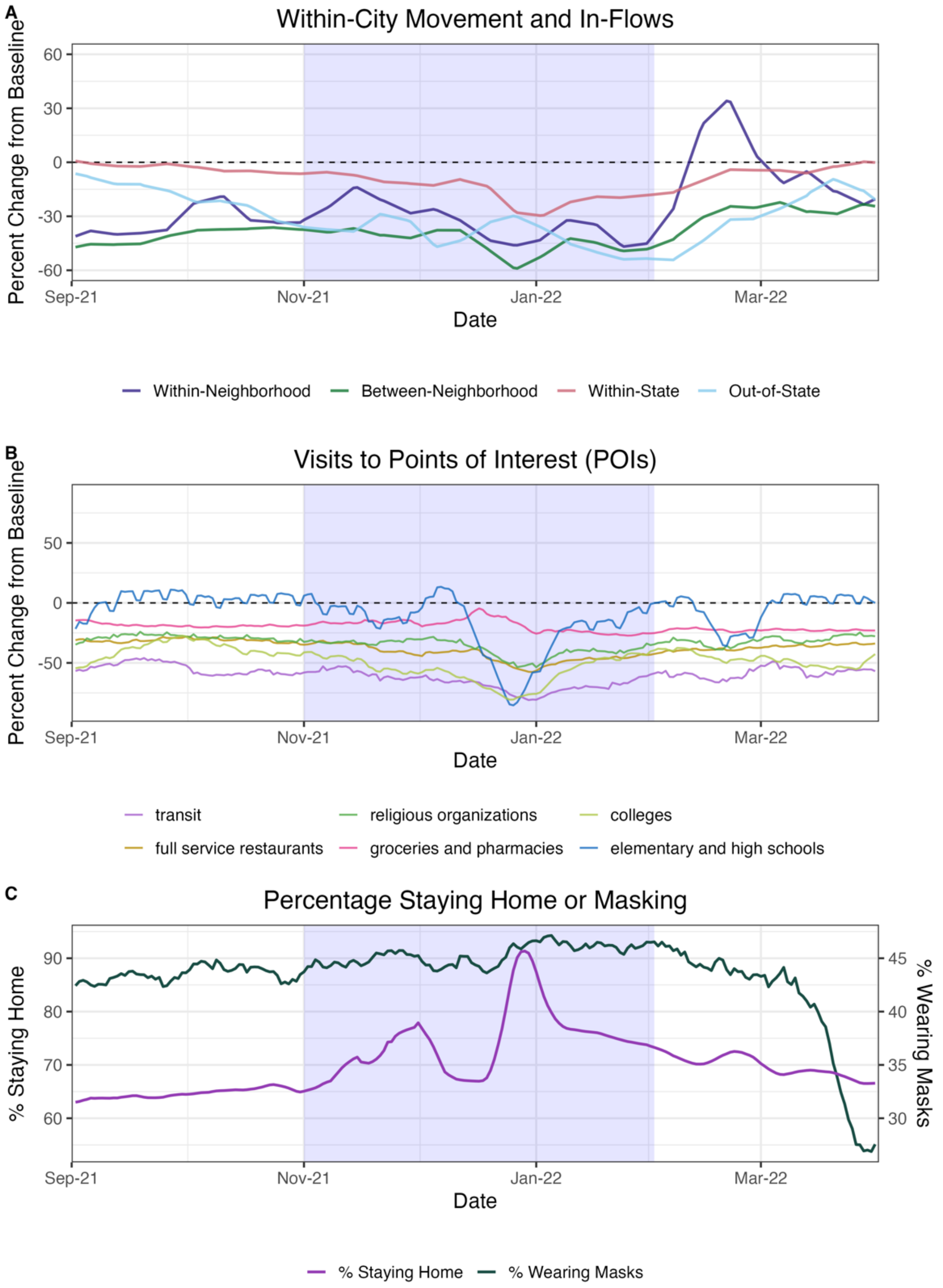

Fig. S17. Mobility and masking trends in the greater Seattle region during November 2021 – April 2022. In each panel, the vertical blue shaded panel indicates the timing of the Omicron BA.1 wave in Seattle (November 2021 – February 2022). **A.** The percent change from baseline for large-scale population movements: within-neighborhood movement (purple), between-neighborhood movement (dark green), inflow of visitors from other Washington counties (red), and inflow of out-of-state visitors (light blue). **B.** The percent change from baseline in foot traffic to various categories of points of interest (POIs): transit stations (purple), religious organizations (green), colleges and universities (light green), full-service restaurants (dark yellow), groceries and pharmacies (pink), and elementary and high schools (blue). **C.** The percentage of devices staying completely at home (purple, left y-axis) and the percentage of individuals masking in public in King County, WA (dark green, right y-axis).

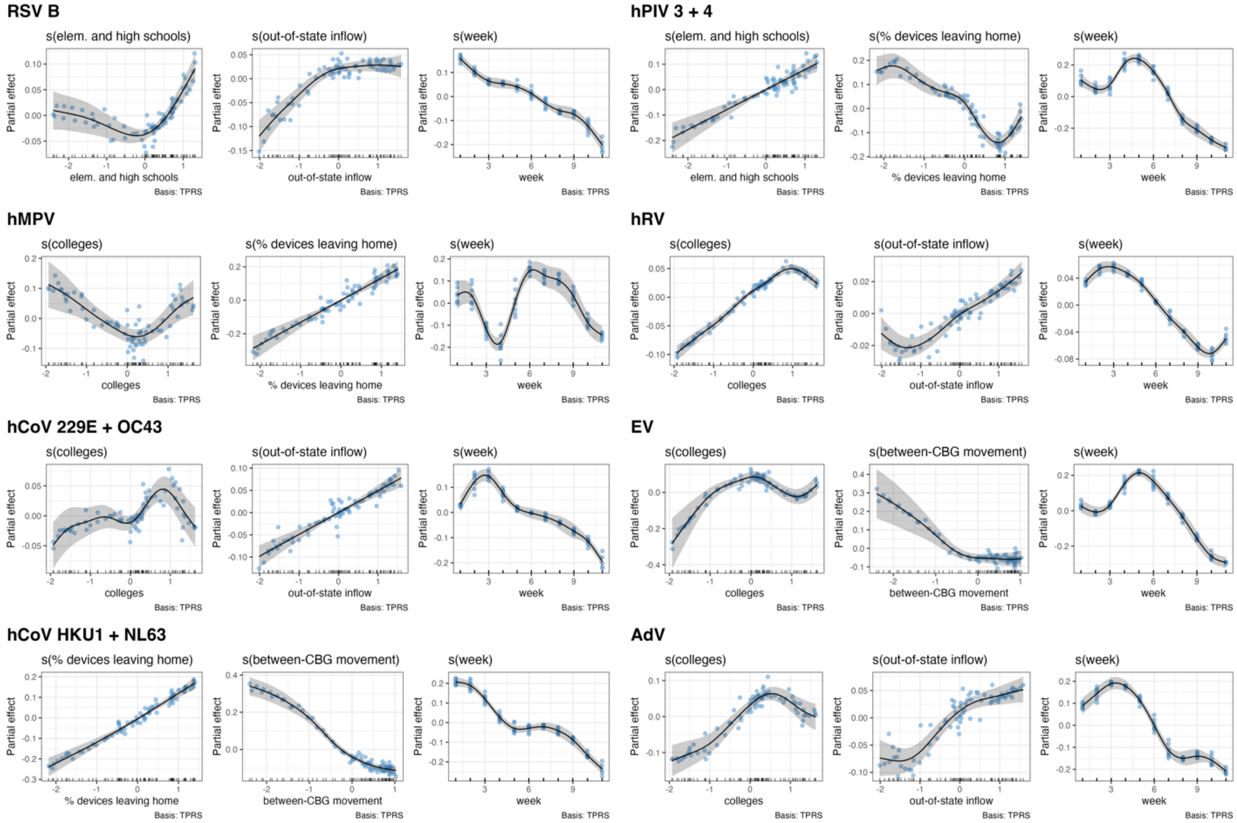

Fig. S18. Generalized additive model (GAM) plots showing the partial effects of selected mobility indicators and time trends on the daily effective reproduction numbers (R_t) of endemic respiratory viruses during the beginning of the Omicron BA.1 wave, November 2021 – January 2022. GAMs were fit to the exponential growth phase of each wave, when R_t exceeds 1. Tick marks on the x-axis indicate observed data points. The y-axis represents of the partial effect of each variable. Gray shaded bands are the 95% confidence intervals of partial effects. The blue points are partial residuals.

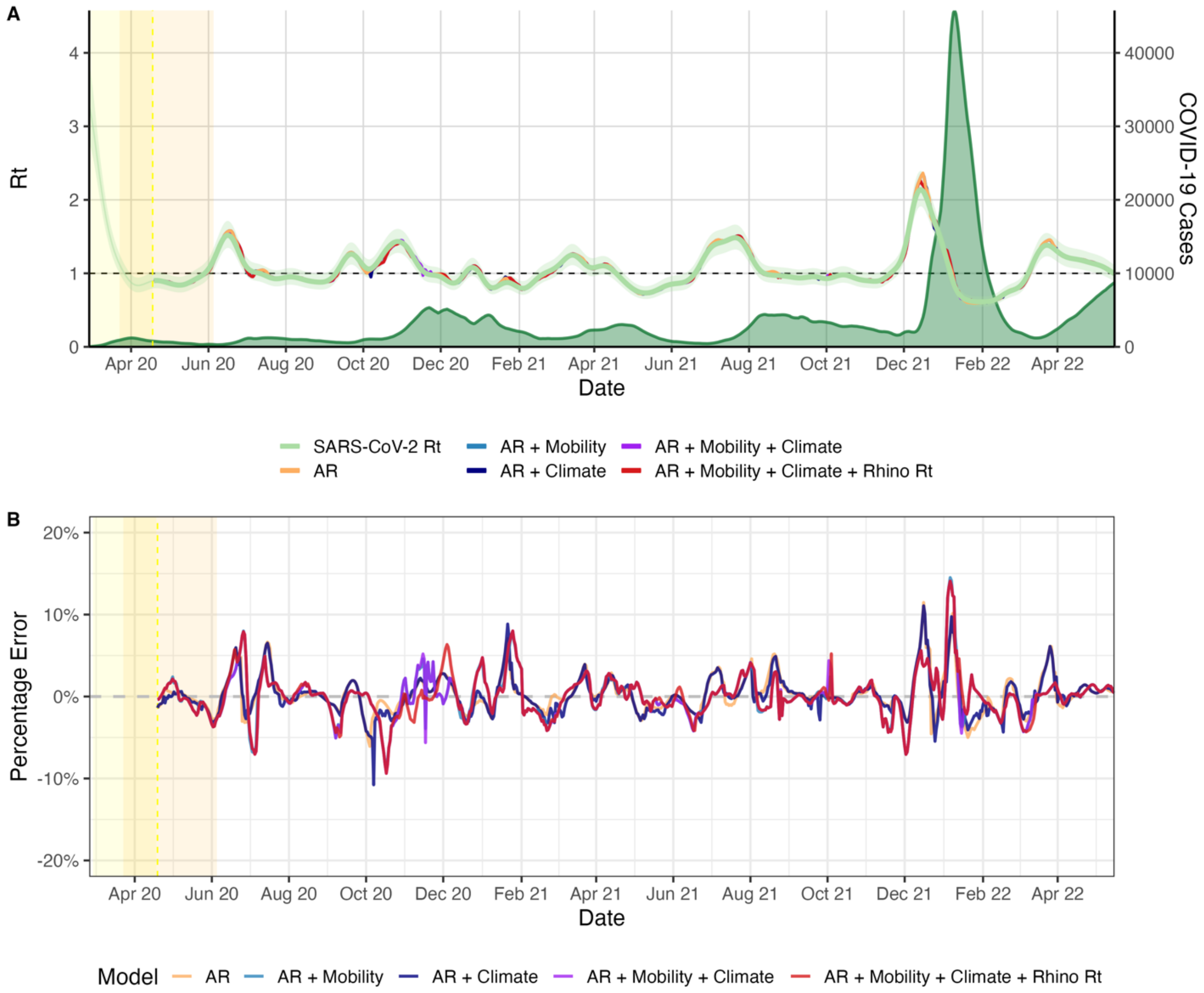

Fig. S19. Predictive model of daily SARS-CoV-2 transmission (time-varying effective reproduction numbers, R_t), March 2020 – May 2022. The vertical yellow shaded area indicates the initial model training period, and the vertical orange shaded area indicates Seattle's stay-at-home period. **A.** Estimated daily transmissibility (R_t) (left y-axis) from the full model with autoregressive (AR) terms and mobility, climatic, and rhinovirus (hRV) interaction covariates (red), contrasting with observed R_t (thick light green line: posterior median; light green shaded band: 90% credible interval) and estimates from a model with only AR terms (orange), a model with AR and mobility terms (light blue), a model with AR and climatic terms (dark blue), and a model with AR, mobility, and climatic terms (purple). Daily COVID-19 cases are shaded dark green (right y-axis). **B.** Model percentage error in predicting R_t. Positive values indicate overprediction, and negative values indicate underprediction.

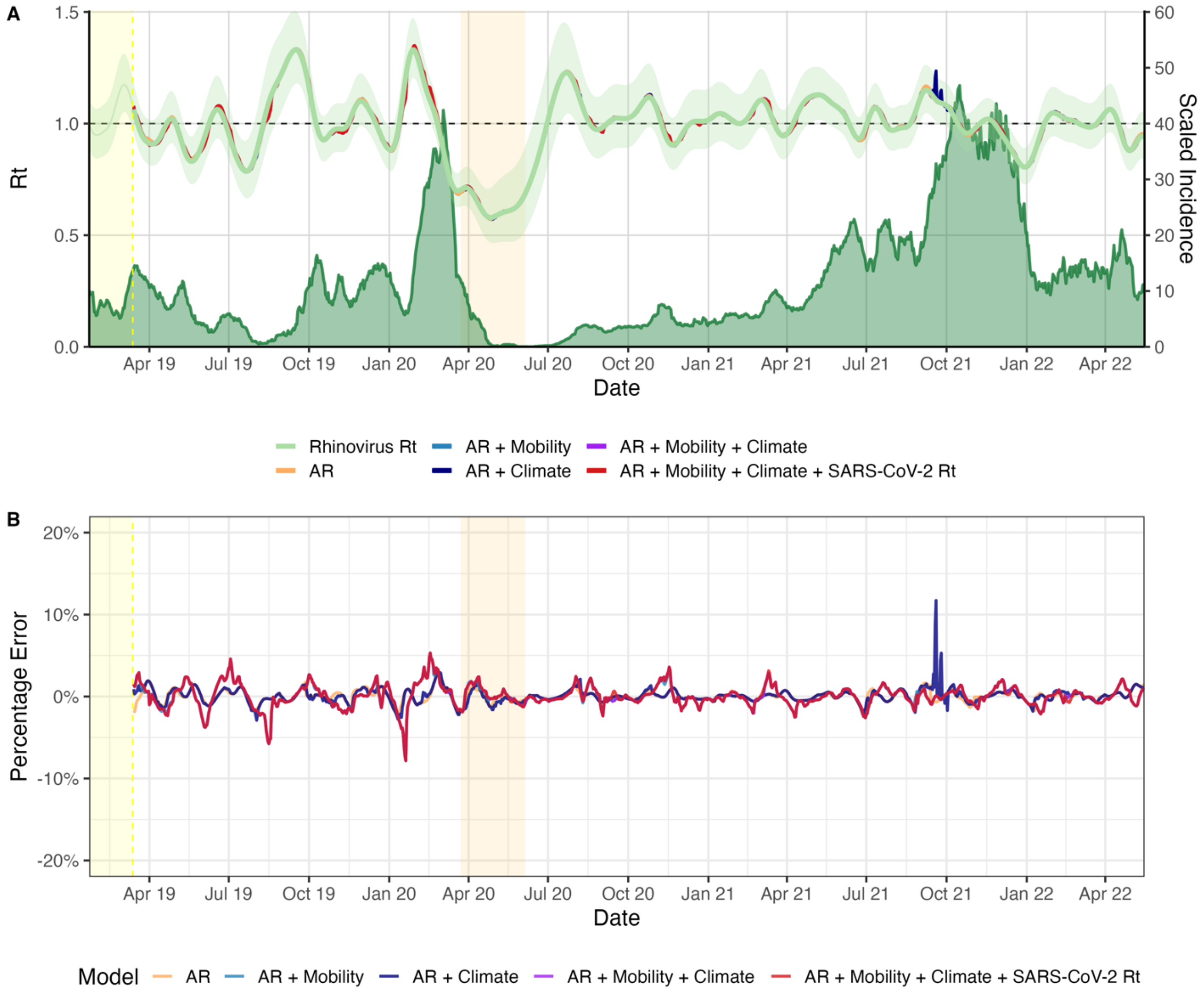

Fig. S20. Predictive model of daily human rhinovirus (hRV) transmission (time-varying effective reproduction numbers, R_t), February 2019 – May 2022. The vertical yellow shaded area indicates the initial model training period, and the vertical orange shaded area indicates Seattle's stay-at-home period. **A.** Estimated daily transmissibility (R_t) (left y-axis) from the full model with autoregressive (AR) terms and mobility, climate, and SARS-CoV-2 interaction covariates (red), contrasting with observed R_t (thick light green line: posterior median; light green shaded band: 90% credible interval) and estimates from a model with only AR terms (orange), a model with AR and mobility terms (light blue), a model with AR and climatic terms (dark blue), and a model with AR, mobility, and climatic terms (purple). Daily hRV incidence is shaded dark green (right y-axis). **B.** Model percentage error in predicting R_t. Positive values indicate overprediction, and negative values indicate underprediction.

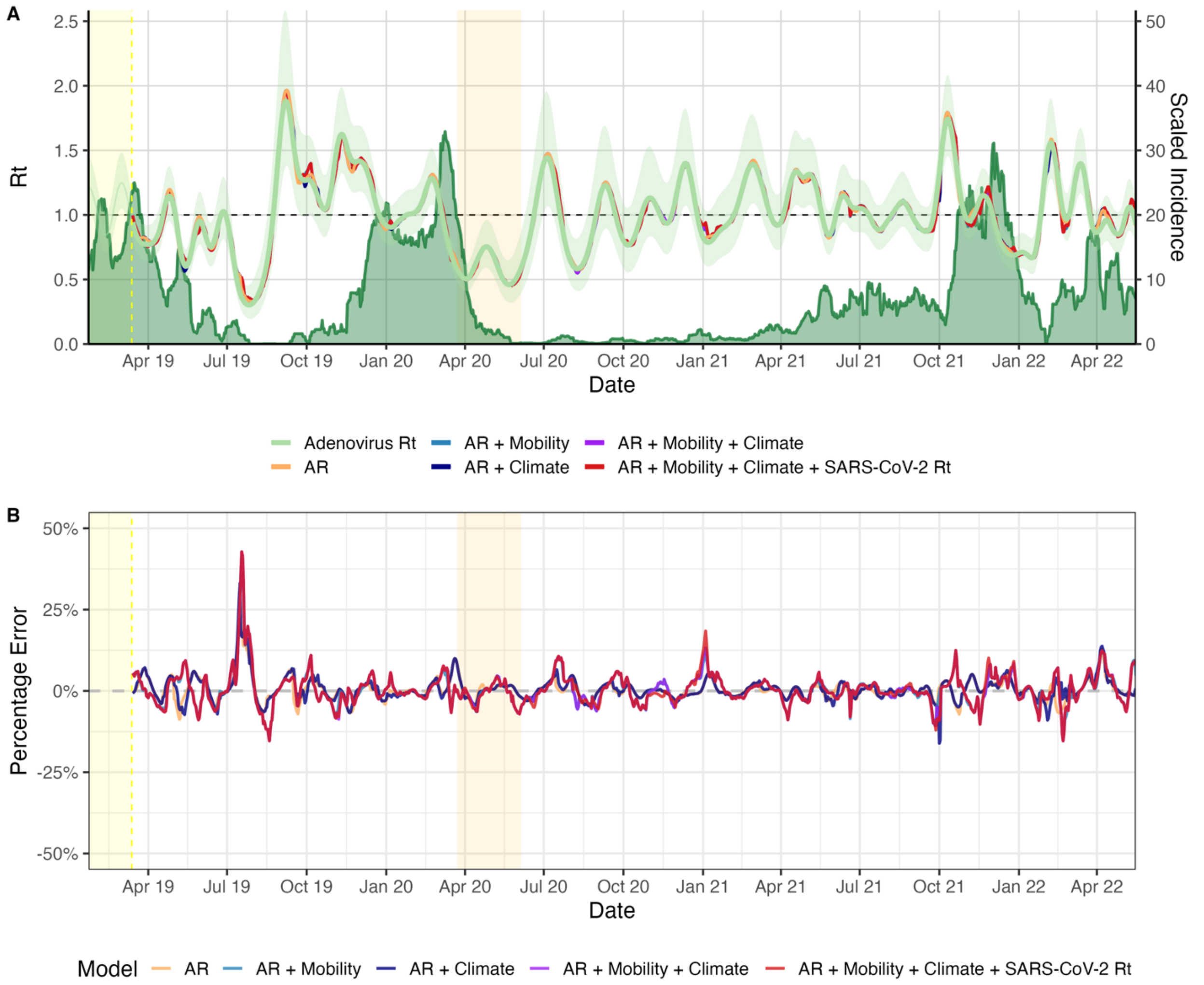

Fig. S21. Predictive model of daily adenovirus (AdV) transmission (time-varying effective reproduction numbers, R_t), February 2019 – May 2022. The vertical yellow shaded area indicates the initial model training period, and the vertical orange shaded area indicates Seattle's stay-at-home period. **A.** Estimated daily transmissibility (R_t) (left y-axis) from the full model with autoregressive (AR) terms and mobility, climate, and SARS-CoV-2 interaction covariates (red), contrasting with observed R_t (thick light green line: posterior median; light green shaded band: 90% credible interval) and estimates from a model with only AR terms (orange), a model with AR and mobility terms (light blue), a model with AR and climatic terms (dark blue), and a model with AR, mobility, and climatic terms (purple). Daily AdV incidence is shaded dark green (right y-axis). **B.** Model percentage error in predicting R_t. Positive values indicate overprediction, and negative values indicate underprediction.

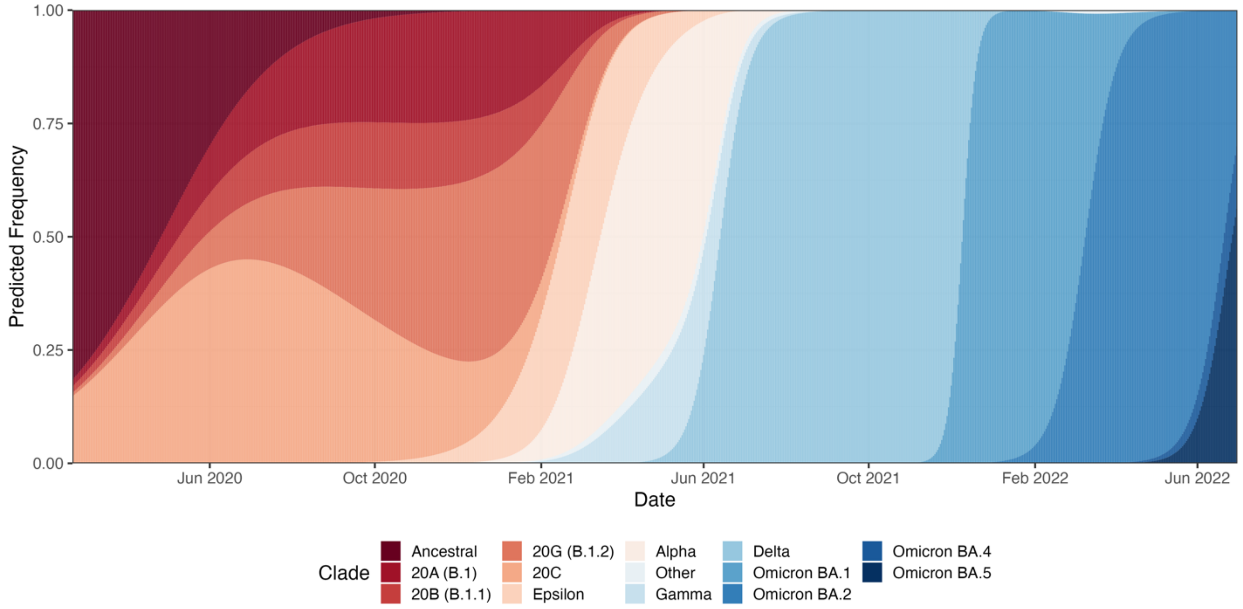

Fig. S22. Daily predicted frequencies of SARS-CoV-2 clades circulating in King County, Washington during February 2020 – June 2022, based on 9,626 genomes. The predicted probabilities of a given sequence belonging to each clade were determined by a multinomial logistic regression model, with SARS-CoV-2 Nextstrain clades as the dependent variable and the number of days since the first detection as the predictor.

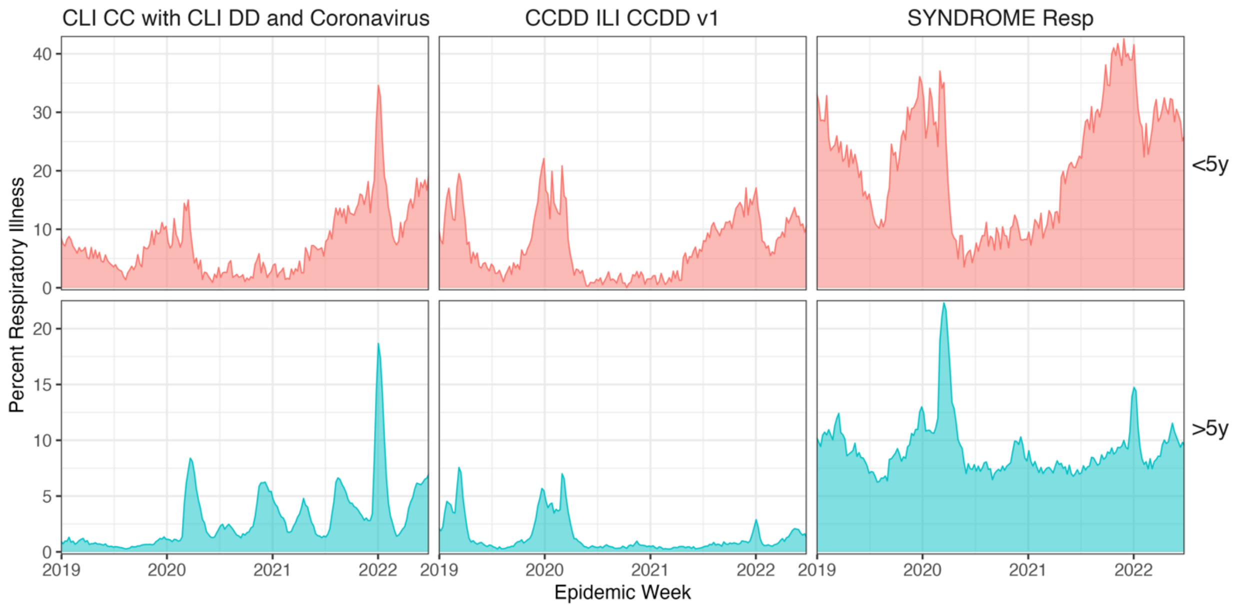

Fig. S23. The weekly proportion of emergency department visits coded as COVID-like illness (CLI), influenza-like illness (ILI), or broad respiratory illness among patients seeking care at emergency departments in King County, Washington. Data are disaggregated by age group: < 5 (top) and ≥ 5 years of age (bottom). We applied centered two-week moving averages to syndromic respiratory illness rates to reduce noise. Respiratory syndromic surveillance data were obtained from the Rapid Health Information Network (RHINO) program at the Washington Department of Health. Syndrome criteria are defined by the Electronic Surveillance System for the Early Notification of Community-Based Epidemics (ESSENCE).

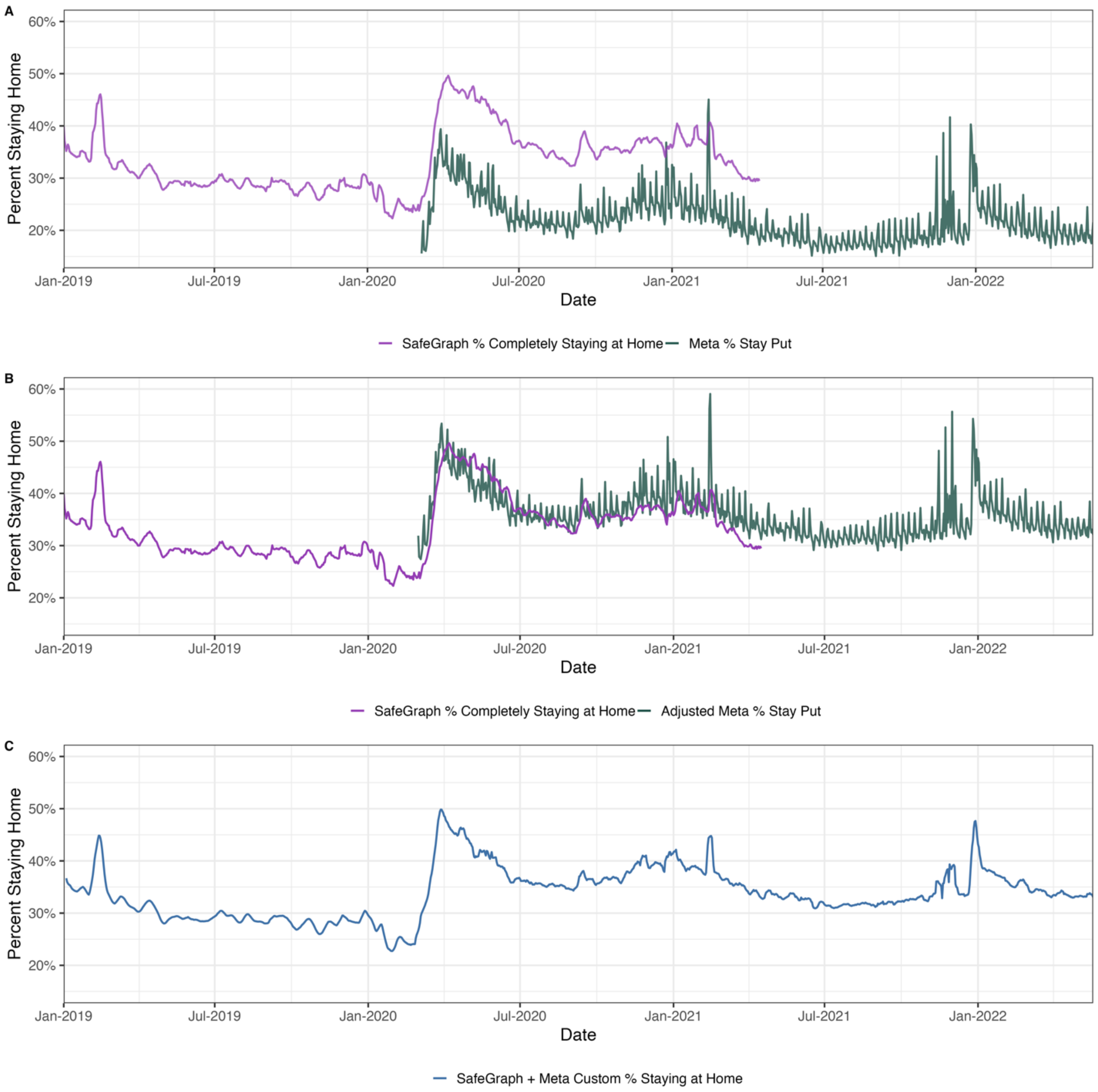

Fig. S24. Combining SafeGraph and Meta Data for Good mobility datasets to create a custom "percentage staying home" metric for King County, Washington. A. Daily data on the percentage of devices staying home in King County from SafeGraph's Social Distancing Metrics (purple) and Meta Data for Good's Movement Range Maps (green). SafeGraph social distancing metrics are available from January 1, 2019, to April 16, 2021, and Meta Movement Range Maps are available from March 1, 2020, to May 22, 2022. Trends in the percentage of devices staying home are almost identical across the two data sources, though the percentage of devices staying home in the Meta dataset is lower than that observed in the SafeGraph dataset. **B.** We added a scaling factor of 14 to the Meta indicator, and **C.** Joined the two time series to create a single metric for our study period (blue). Because the Meta data are much noisier than the SafeGraph data, we smoothed the joint time series with a centered 7-day moving average.

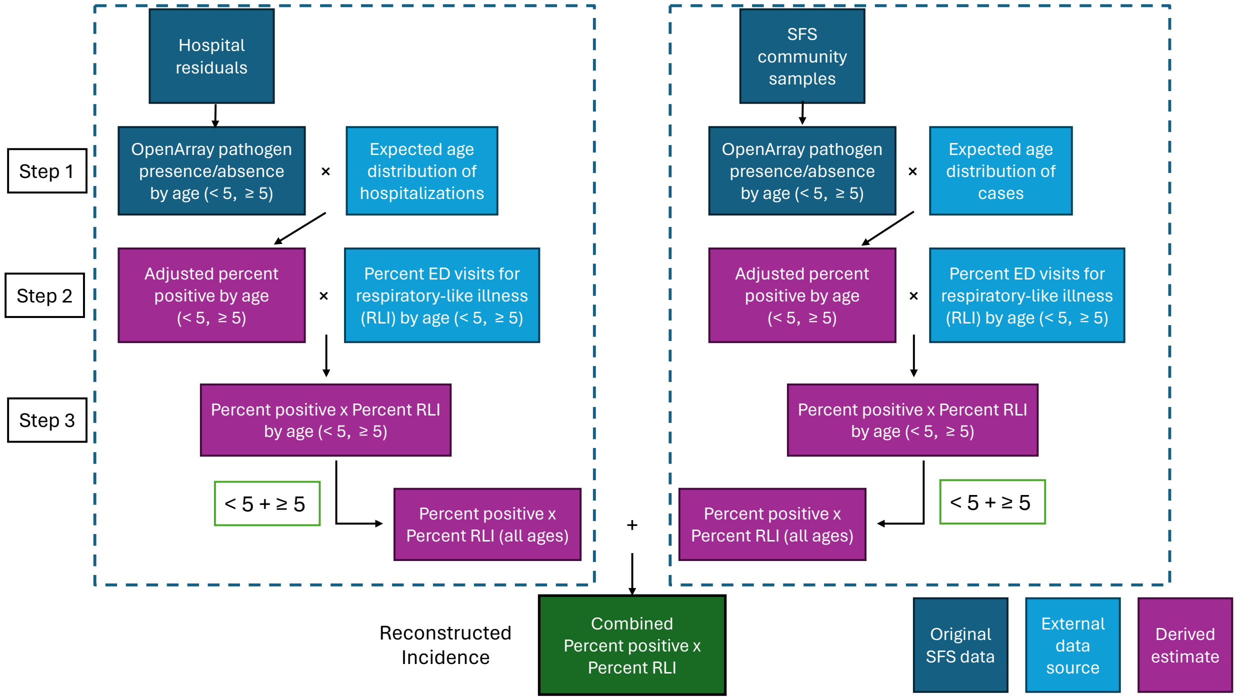

Fig. S25. Flow chart showing the three steps for reconstructing pathogen incidences from virologic and syndromic respiratory surveillance data. To properly reconstruct pathogen incidences through time, we considered the different populations sampled by the Seattle Flu Study (SFS), particularly regarding age group (≥ 5 years or < 5 years) and clinical setting (hospital or community). Dark blue boxes correspond to data collected by SFS, light blue boxes correspond to external data sources, purple boxes correspond to derived estimates, and the green box is the final output: reconstructed incidence.

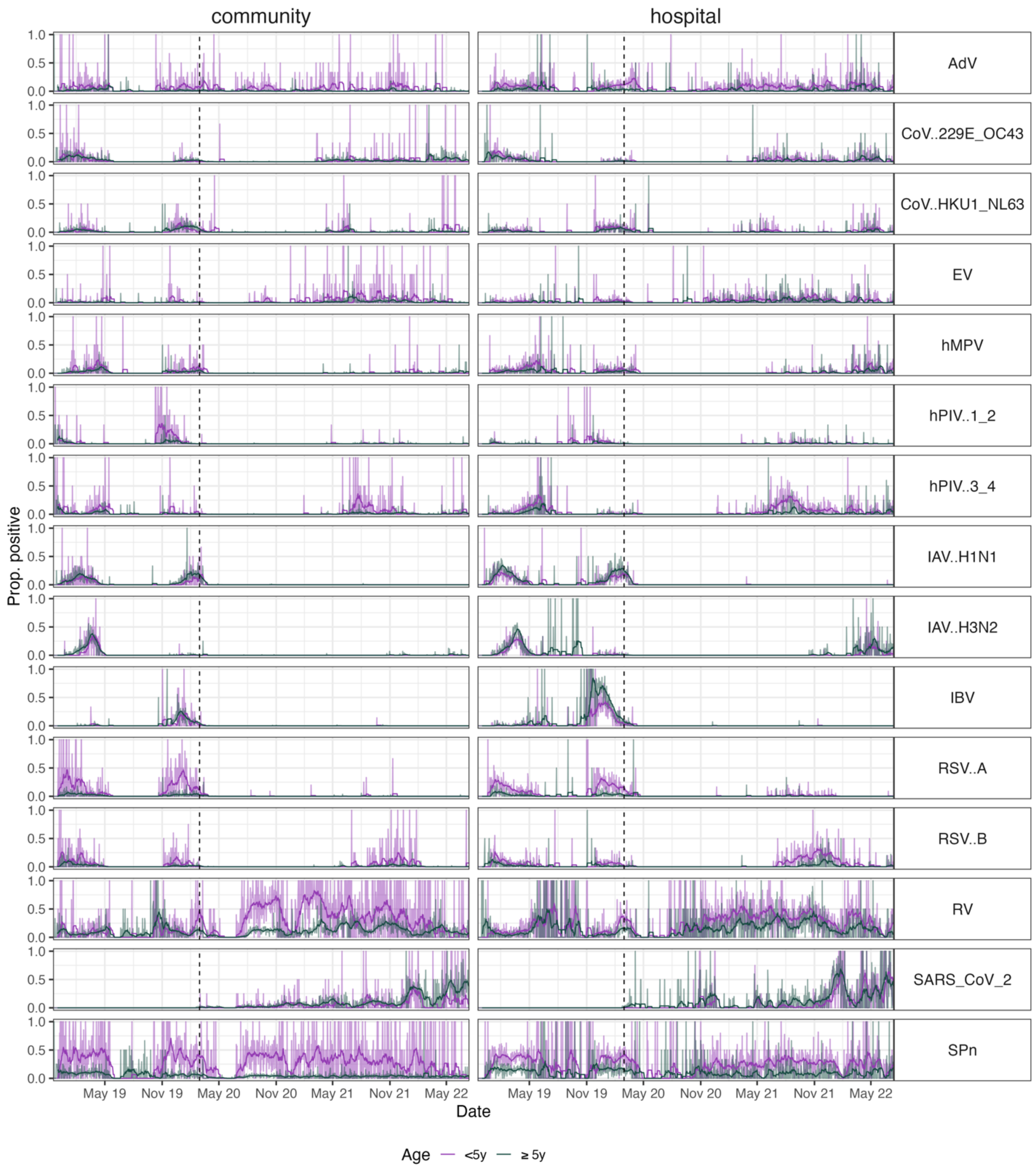

Fig. S26. Daily proportions of respiratory specimens testing positive for each pathogen during November 2018 - June 2022. Proportion positive values are disaggregated by clinical setting (community or hospital) and age group (≥ 5 years, green or < 5 years, purple). Raw proportion positive values are overlaid with centered two-week moving averages.

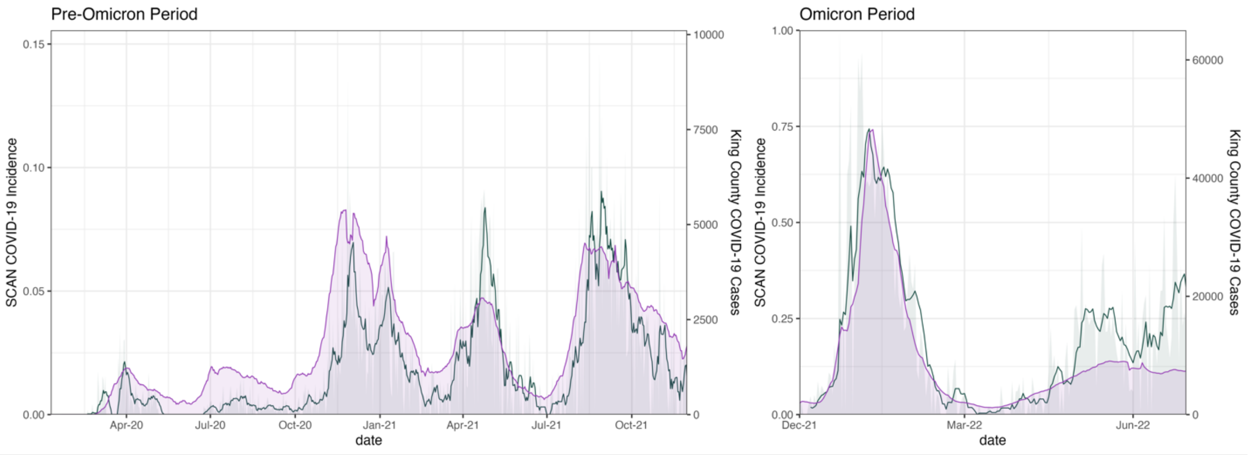

Fig. S27. Comparison of SCAN estimated daily COVID-19 incidence (green) to daily confirmed COVID-19 cases (purple) reported in King County, Washington. Comparisons are split into two time periods: pre-Omicron (before December 2021) and Omicron, due to high case counts during the Omicron BA.1 wave in winter 2021-2022. We applied a centered two-week moving average to each time series to reduce noise.

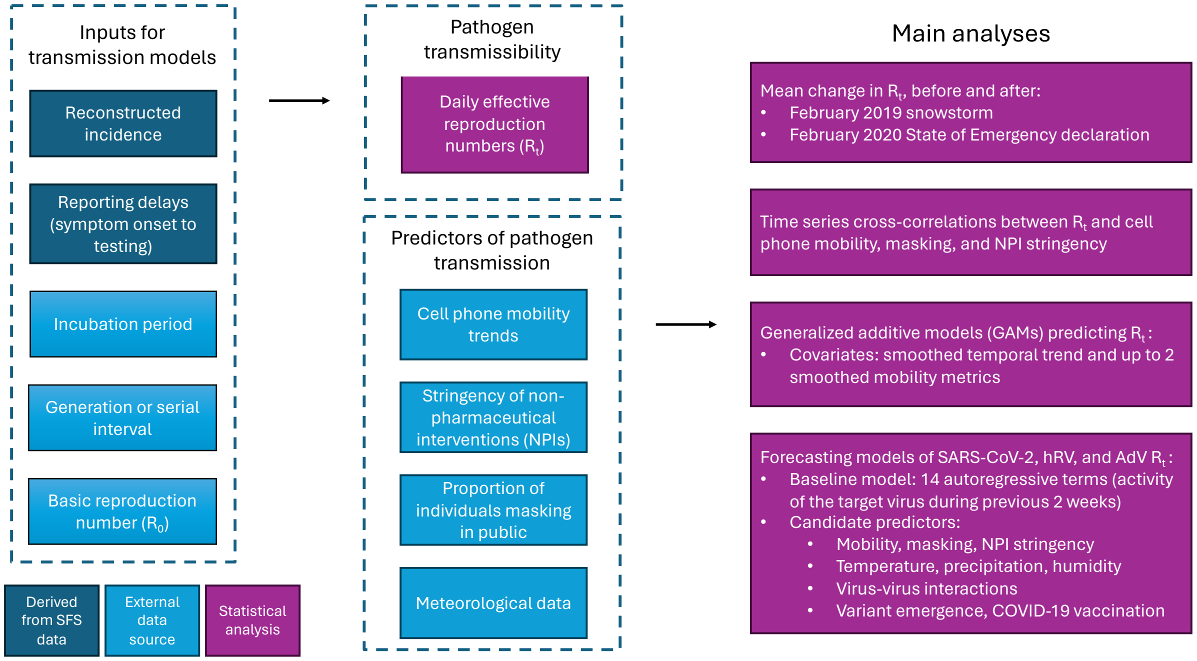

Fig. S28. An overview of statistical analyses and their various inputs. Dark blue boxes correspond to inputs derived from Seattle Flu Study (SFS) data, light blue boxes correspond to inputs from external data sources, and purple boxes correspond to statistical analyses.

Supplementary Tables

Table S1. TaqMan OpenArray panel probe sets over time. Columns V1-V5 represent different probe sets. Date ranges correspond to when each probe set was in use and not specimen collection dates. Respiratory specimen collection began in November 2018, and laboratory testing of specimens began in March 2019.

Pathogen type	Probe	**V1** Start: 3/12/19 End: 4/16/19	**V2** Start: 5/5/19 End: 2/20/20	**V3** Start: 2/21/20 End: 5/1/20	**V4** Start: 5/29/20 End: 11/20/20	**V5** Start: 11/23/20 End: ongoing
**Influenza virus**	Flu_A_pan					
	Flu_A_H1					
	Flu_A_H3					
	Flu_B_pan					
	Flu_C					
**Parainfluenza virus**	hPIV1					
	hPIV2					
	hPIV3					
	hPIV4					
	hPIV1_hPIV2					
	hPIV3_hPIV4					
**Enterovirus**	EV_pan					
	EV_D68					
**Rhinovirus**	RV_1of1					
	RV_1of2					
**Adenovirus**	AdV_1of1					
	AdV_1of2					
**Coronavirus**	CoV_HKU1_CoV_NL63					
	CoV_229E_CoV_OC43					
	hCoV_HKU1					
	hCoV_NL63					
	hCoV_229E					
	hCoV_OC43					
	SARS_CoV-2_Orf1B					
	SARS-CoV-2_S					
**Respiratory syncytial virus**	RSVA					
	RSVB					
**Metapneumovirus**	hMPV					
**Parechovirus**	hPeV					
**Bocavirus**	hBoV					
**Measles**	Measles					
**Mumps**	Mumps					
**Pneumoniae**	M. pneumoniae					
	C. pneumoniae					
	S. pneumoniae					
**Total unique pathogens**		**24**	**26**	**26**	**24**	**24**

Table S2. Number of samples by recruitment type and site.

Recruitment Type	Site	Sample size
Clinic (Kiosk)	ChildrensHospitalSeattle	944
Clinic (Kiosk)	ChildrensHospitalSeattleOutpatientClinic	246
Clinic (Kiosk)	UWHallHealth	196
Clinic (Kiosk)	ChildrensHospitalBellevue	94
Clinic (Kiosk)	UWSeaMar	88
Clinic (Kiosk)	PioneerSquare	58
Clinic (Kiosk)	ChildrensSeaMar	37
Clinic (Flu VE Network)	Kaiser Permanente	3604
Community (swab-and-send)	SCAN	42840
Community (swab-and-send)	swabNSend	2901
Community (Residual)	RetrospectivePHSKC	7956
Community (Kiosk)	WestlakeMall	392
Community (Kiosk)	CapitolHillLightRailStation	32
Community (Kiosk)	SeattleCenter	23
Community (Kiosk)	SeaTacDomestic	12
Community (Kiosk)	SeaTacInternational	9
Community (Kiosk)	PICAWA	6
Community (Kiosk)	KingStreetStation	4
Community (Kiosk)	WestlakeLightRailStation	1
College Campus (Kiosk)	UWSuzzalloLibrary	177
College Campus (Kiosk)	HUB	135
Workplace (Kiosk)	HarborviewLobby	254
Workplace (Kiosk)	FredHutchLobby	171
Workplace (Kiosk)	Costco	34
Workplace (Kiosk)	ColumbiaCenter	18
Hospital (Residual)	RetrospectiveChildrensHospitalSeattle	16042
Hospital (Residual)	RetrospectiveHarborview	2483
Hospital (Residual)	RetrospectiveNorthwest	1426
Hospital (Residual)	RetrospectiveUWMedicalCenter	708
Total samples		80891

Table S3. Comparison of different models forecasting daily effective reproduction numbers (R_t) of human rhinovirus (hRV), adenovirus (AdV), and SARS-CoV-2 over the course of the study period. The accuracy of 7-day ahead forecasts were measured using the root-mean-squared error (RMSE) and mean absolute error (MAE). The benefit of including variables related to mobility, climate, or viral interactions was calculated as the percent difference in RMSE and MAE relative to the baseline AR model, wherein negative values indicate models with additional covariates are more accurate, and positive values indicate the baseline model is more accurate.

**Human rhinovirus (hRV)**<br>First training window: 2019 February 6 – 2019 March 7<br>Testing period: 2019 March 8 – 2022 May 16	**RMSE**	**RMSE**<br>**Percent difference from baseline**	**MAE**	**MAE**<br>**Percent difference from baseline**
AR	**0.0069**	**0**	**0.0051**	**0**
AR + Climate	0.0091	32.7	0.0059	15.5
AR + Mobility	0.0133	93.9	0.0092	81.4
AR + Mobility + Climate	0.0135	97.4	0.0094	85.3
AR + Mobility + Climate + SARS-CoV-2 R_t	0.0136	97.6	0.0094	85.6

**Adenovirus (AdV)**<br>First training window: 2019 February 6 – 2019 March 7<br>Testing period: 2019 March 8 – 2022 May 16	**RMSE**	**RMSE**<br>**Percent difference from baseline**	**MAE**	**MAE**<br>**Percent difference from baseline**
AR	**0.0290**	**0**	**0.0205**	**0**
AR + Climate	0.0313	8.0	0.0221	7.9
AR + Mobility	0.0402	38.7	0.0301	46.7
AR + Mobility + Climate	0.0414	43.0	0.0310	51.4
AR + Mobility + Climate + SARS-CoV-2 R_t	0.0424	46.4	0.0315	53.8

**SARS-CoV-2**<br>First training window: 2020 March 14 – 2020 April 12<br>Testing period: 2020 April 13 – 2022 May 16	**RMSE**	**RMSE**<br>**Percent difference from baseline**	**MAE**	**MAE**<br>**Percent difference from baseline**
AR	**0.0291**	**0**	**0.0175**	**0**
AR + Climate	0.0292	0.4	0.0179	2.5
AR + Mobility	0.0295	1.4	0.0194	11.2
AR + Mobility + Climate	0.0298	2.4	0.0197	12.7
AR + Mobility + Climate + hRV R_t	0.0302	3.9	0.0199	14.1

Table S4. Comparison of different models forecasting daily effective reproduction numbers (R_t) of human rhinovirus (hRV), adenovirus (AdV), and SARS-CoV-2 during COVID-19 stay-at-home orders and the initial lifting of restrictions. The accuracy of 7-day ahead forecasts were measured using the root-mean-squared error (RMSE) and mean absolute error (MAE). The benefit of including variables related to mobility, climate, or viral interactions was calculated as the percent difference in RMSE and MAE relative to the baseline AR model, wherein negative values indicate models with additional covariates are more accurate, and positive values indicate the baseline model is more accurate.

**Human rhinovirus (hRV)**<br>Testing period: 29 February 2020 – 15 June 2020	**RMSE**	**RMSE Percent difference from baseline**	**MAE**	**MAE Percent difference from baseline**
AR	**0.0057**	**0**	**0.0042**	**0**
AR + Climate	0.0062	8.4	0.0049	16.6
AR + Mobility	0.0064	11.8	0.0050	19.7
AR + Mobility + Climate	0.0066	16.9	0.0052	24.3
AR + Mobility + Climate + SARS-CoV-2 R_t	0.0067	17.4	0.0052	24.9

**Adenovirus (AdV)**<br>Testing period: 29 February 2020 – 15 June 2020	**RMSE**	**RMSE Percent difference from baseline**	**MAE**	**MAE Percent difference from baseline**
AR	0.0193	0	0.0141	0
AR + Climate	**0.0192**	**-0.5**	**0.0138**	**-2.2**
AR + Mobility	0.0216	12.2	0.0168	19.5
AR + Mobility + Climate	0.0236	22.7	0.0181	28.5
AR + Mobility + Climate + SARS-CoV-2 R_t	0.0252	30.7	0.0194	37.5

**SARS-CoV-2**<br>Testing period: 13 April 2020 – 15 June 2020	**RMSE**	**RMSE Percent difference from baseline**	**MAE**	**MAE Percent difference from baseline**
AR	0.0256	0	**0.0162**	**0**
AR + Climate	0.0264	3.0	0.0167	3.6
AR + Mobility	0.0218	-14.8	0.0170	5.4
AR + Mobility + Climate	**0.0218**	**-14.9**	0.0170	5.5
AR + Mobility + Climate + hRV R_t	0.0268	4.5	0.0192	19.1

Table S5. Comparison of different models forecasting daily effective reproduction numbers (R_t) of SARS-CoV-2 during the period of COVID-19 vaccination and SARS-CoV-2 variant emergence, 2021 - 2022. The accuracy of 7-day ahead forecasts were measured using the root-mean-squared error (RMSE) and mean absolute error (MAE). The benefit of including variables related to mobility, climate, or viral interactions was calculated as the percent difference in RMSE and MAE relative to the baseline model (model with only AR terms), wherein negative values indicate models with additional covariates are more accurate, and positive values indicate the baseline model is more accurate.

**SARS-CoV-2**<br>First training window: 2021 January 29 – 2021 February 27<br>Testing period: 2021 February 28– 2022 May 16	**RMSE**	**RMSE**<br>**Percent difference from baseline**	**MAE**	**MAE**<br>**Percent difference from baseline**
AR	0.0334	0	0.0191	0
AR + Climate	0.0323	-3.3	0.0182	-4.9
AR + Mobility	**0.0290**	**-13.2**	**0.0176**	**-7.6**
AR + Mobility + Climate	0.0291	-13.0	0.0178	-6.5
AR + Mobility + Climate + hRV R_t	0.0293	-12.5	0.0179	-6.1
AR + Mobility + Climate + hRV R_t + Vaccination + Variants	0.0330	-1.3	0.0191	0.1

Table S6. Data sources for adjusting the age distributions of pathogen presence/absence data.

Pathogen	Source	Description	Proportion < 5 years	Proportion ≥ 5 years
**Community samples**				
Influenza A/H3N2, A/H1N1, B	[6]	Age group distribution of influenza positive specimens reported by public health laboratories in WA state	Time varying (weekly)	Time varying (weekly)
AdV, hCoV, hMPV, hPIV, RSV, hRV, EV	[6]	Age group distribution of influenza-like illness cases in WA state	Time varying (weekly)	Time varying (weekly)
SARS-CoV-2	[25]	Age group distribution of COVID-19 positive specimens in King County, WA	Time varying (daily)	Time varying (daily)
**Hospital residuals**				
Influenza A/H3N2, A/H1N1, B	[26]	National age group distribution of laboratory-confirmed influenza-associated hospitalizations	Time varying (weekly)	Time varying (weekly)
RSV	[27]	Age distribution of RSV detections among 186,155 positive patients hospitalized for ARI, cardiorespiratory disease, or sepsis, United States, 1997-2009	0.54	0.46
hRV, EV	[28]	Age distribution of hRV detections among 76 positive patients hospitalized for ARI, Taiwan, 2013-2014	0.64	0.36
AdV	[29]	Age distribution of AdV detections among 1302 positive patients referred to the Institute for Infectious Diseases for diagnostic testing, Bern, Switzerland, 1998-2017	0.57	0.43
hCoV	[30]	Age distribution of hCoV detections among 2958 positive patients in secondary care, NHS Greater Glasgow and Clyde, Scotland, UK, 2005-2017	0.29	0.71
hMPV	[31]	Age distribution of hMPV detections among 331 positive patients hospitalized for SARI, Mexico, 2009-2018	0.575	0.425
hPIV	[32]	Age distribution of hPIV detections among 17,717 positive patients in primary or secondary care, England and Wales, UK, 1998-2013	0.64	0.36
SARS-CoV-2	[25]	Age group distribution of laboratory confirmed COVID-19 hospitalizations in Washington state	Time varying (daily)	Time varying (daily)

Table S7. Pathogen-specific incubation periods, generation or serial intervals, and basic reproduction numbers obtained from published literature. Incubation periods and generation or serial intervals include the mean and standard deviation (SD) in days. The probability distribution family used to estimate each parameter is listed below the mean and SD.

Pathogen	Incubation Period (days)	Generation or Serial Interval (days)	Basic Reproduction Number, R_o	Source
SARS-CoV-2	Mean = 6.3, SD = 3.6<br>Lognormal	Mean = 5.2, SD = 1.2<br>Gamma	3	[33-35]
hCoV*	Mean = 5.1, SD = 2.2<br>Lognormal	Mean = 5.2, SD = 1.2<br>Gamma	3	[34,36]
Influenza	Mean = 1.9, SD = 1.22<br>Lognormal	Mean = 3.6, SD = 1.6<br>Weibull	1.2 (A/H3N2 and B)<br>1.5 (A/H1N1)	[37-39]
RSV†	Mean = 4.5, SD = 0.9<br>Lognormal	Mean = 7.5, SD = 2.1<br>Gamma	2	[36,40-42]
hMPV†‡	Mean = 4.5, SD = 0.9<br>Lognormal	Mean = 5.2, SD = 1.5<br>Gamma	2	[36,42,43]
hPIV†§	Mean = 2.6, SD = 1.35<br>Lognormal	Mean = 7.5, SD = 2.1<br>Gamma	2	[37,40,41]
hRV†	Mean = 2.36, SD = 1.1<br>Lognormal	Mean = 4.4, SD = 2.7<br>Gamma	2	[36,41,44]
EV¶	Mean = 2.36, SD = 1.1<br>Lognormal	Mean = 4.4, SD = 2.7<br>Gamma	2	[36,41,44]
AdV†	Mean = 5.6, SD = 1.26<br>Lognormal	Mean = 7.8, SD = 2.4<br>Gamma	2	[37,41,45]

* Generation interval and R_o for SARS-CoV-2.

† Serial interval reanalyzed using time intervals of disease onset for infectors and infectees from the published study.

‡ Incubation period and R_o for RSV.

§ Serial interval for RSV.

¶ Incubation period, serial interval, and R_o for hRV.

Table S8. International Classification of Diseases, Tenth Revision (ICD-10) codes used to designate hospitalized patients as symptomatic for respiratory illness.

Condition	ICD-10 Code
Acute upper respiratory infections	J00-J06
Influenza and pneumonia	J10-J18
Other acute lower respiratory infections	J20-J22
Other diseases of upper respiratory tract	J30-J39
Chronic lower respiratory diseases	J40-J47
Other respiratory diseases principally affecting the interstitium	J80-J84
Suppurative and necrotic conditions of the lower respiratory tract	J85-J86
Other diseases of the pleura	J90-J94
Other diseases of the respiratory system	J96-J99
COVID-19	U07.1
Otitis media	H65-H66
Hemorrhage from respiratory passages	R04
Cough	R05
Abnormalities in breathing	R06
Pain in throat and chest	R07
Hypoxemia	R09.02
Nasal congestion or postnasal drip	R09.8
Fever, unspecified	R50.9
Respiratory tuberculosis	A15
Viral infection of unspecified site	B34
Viral conjunctivitis	B30
Streptococcus, Staphylococcus, and Enterococcus as the cause of diseases classified elsewhere	B95
Adenovirus as the cause of diseases classified elsewhere	B97.0
Enterovirus as the cause of diseases classified elsewhere	B97.1
Coronavirus as the cause of diseases classified elsewhere	B97.2
Respiratory syncytial virus as the cause of diseases classified elsewhere	B97.4
Human metapneumovirus as the cause of diseases classified elsewhere	B97.81

Supplementary References

1 Jackson, M. L. *et al.* Incidence of Medically Attended Acute Respiratory Illnesses Due to Respiratory Viruses Across the Life Course During the 2018/19 Influenza Season. *Clin Infect Dis* **73**, 802-807 (2021). https://doi.org/10.1093/cid/ciab131
2 Jackson, M. L. *et al.* Influenza Vaccine Effectiveness in the United States during the 2015-2016 Season. *N Engl J Med* **377**, 534-543 (2017). https://doi.org/10.1056/NEJMoa1700153
3 Kim, A. E. *et al.* Evaluating Specimen Quality and Results from a Community-Wide, Home-Based Respiratory Surveillance Study. *J Clin Microbiol* **59** (2021). https://doi.org/10.1128/JCM.02934-20
4 Chung, E. *et al.* Comparison of Symptoms and RNA Levels in Children and Adults With SARS-CoV-2 Infection in the Community Setting. *JAMA Pediatr* **175**, e212025 (2021). https://doi.org/10.1001/jamapediatrics.2021.2025
5 Hansen, C. *et al.* Trends in Risk Factors and Symptoms Associated With SARS-CoV-2 and Rhinovirus Test Positivity in King County, Washington, June 2020 to July 2022. *JAMA Network Open* **5**, e2245861 (2022). https://doi.org/10.1001/jamanetworkopen.2022.45861
6 National Center for Immunization and Respiratory Diseases (NCIRD), U.S. Centers for Disease Control and Prevention. *FluView Interactive*, https://www.cdc.gov/flu/weekly/fluviewinteractive.htm. Accessed: 2023-10-20 (2023).
7 Rudis, B. cdcfluview: Retrieve Flu Season Data from the United States Centers for Disease Control and Prevention ('CDC') 'FluView' Portal. R package version 0.9.4 (2021).
8 Carpenter, B. *et al.* Stan: A Probabilistic Programming Language. *Journal of Statistical Software* **76**, 1 - 32 (2017). https://doi.org/10.18637/jss.v076.i01
9 Abbott, S. *et al.* Estimating the time-varying reproduction number of SARS-CoV-2 using national and subnational case counts. *Wellcome Open Research* **5** (2020). https://doi.org/10.12688/wellcomeopenres.16006.1
10 Scott, J. A. *et al.* Epidemia: An R package for semi-mechanistic bayesian modelling of infectious diseases using point processes. *arXiv preprint arXiv:2110.12461* (2021). https://doi.org/10.48550/arXiv.2110.12461
11 Bhatt, S. *et al.* Semi-mechanistic Bayesian modelling of COVID-19 with renewal processes. *Journal of the Royal Statistical Society Series A: Statistics in Society* **186**, 601-615 (2023). https://doi.org/10.1093/jrsssa/qnad030
12 Cori, A., Ferguson, N. M., Fraser, C. & Cauchemez, S. A new framework and software to estimate time-varying reproduction numbers during epidemics. *Am J Epidemiol* **178**, 1505-1512 (2013). https://doi.org/10.1093/aje/kwt133
13 Gostic, K. M. *et al.* Practical considerations for measuring the effective reproductive number, Rt. *PLoS Comput Biol* **16**, e1008409 (2020). https://doi.org/10.1371/journal.pcbi.1008409
14 Huisman, J. S. *et al.* Estimation and worldwide monitoring of the effective reproductive number of SARS-CoV-2. *Elife* **11** (2022). https://doi.org/10.7554/eLife.71345
15 Hoffman, M. D. & Gelman, A. The No-U-Turn sampler: adaptively setting path lengths in Hamiltonian Monte Carlo. *J. Mach. Learn. Res.* **15**, 1593-1623 (2014).
16 Goldstein, E. *et al.* Reconstructing influenza incidence by deconvolution of daily mortality time series. *Proceedings of the National Academy of Sciences* **106**, 21825-21829 (2009). https://doi.org/10.1073/pnas.0902958106
17 Wood, S. N. Fast stable restricted maximum likelihood and marginal likelihood estimation of semiparametric generalized linear models. *Journal of the Royal Statistical Society: Series B (Statistical Methodology)* **73**, 3-36 (2011). https://doi.org/10.1111/j.1467-9868.2010.00749.x
18 Simpson, G. L. Modelling Palaeoecological Time Series Using Generalised Additive Models. *Frontiers in Ecology and Evolution* **6** (2018). https://doi.org/10.3389/fevo.2018.00149
19 Yang, S., Santillana, M. & Kou, S. C. Accurate estimation of influenza epidemics using Google search data via ARGO. *Proc Natl Acad Sci U S A* **112**, 14473-14478 (2015). https://doi.org/10.1073/pnas.1515373112
20 National Centers for Environmental Information. *U.S. Local Climatological Database*, https://www.ncei.noaa.gov/products/land-based-station/local-climatological-data. Accessed: 2023-01-30 (2023).
21 Public Health - Seattle & King County. *COVID-19 vaccination among King County residents*, https://kingcounty.gov/en/dept/dph/health-safety/disease-illness/covid-19/data/vaccination. Accessed: 2024-01-03 (2024).

22 Hadfield, J. *et al.* Nextstrain: real-time tracking of pathogen evolution. *Bioinformatics* **34**, 4121-4123 (2018). https://doi.org/10.1093/bioinformatics/bty407
23 Khare, S. *et al.* GISAID's Role in Pandemic Response. *China CDC Wkly* **3**, 1049-1051 (2021). https://doi.org/10.46234/ccdcw2021.255
24 Delussu, F., Tizzoni, M. & Gauvin, L. The limits of human mobility traces to predict the spread of COVID-19: A transfer entropy approach. *PNAS Nexus* **2**, pgad302 (2023). https://doi.org/10.1093/pnasnexus/pgad302
25 Washington State Department of Health. *COVID-19 Data Dashboard*, https://doh.wa.gov/emergencies/covid-19/data-dashboard. Accessed: 2023-02-21 (2023).
26 Chaves, S. S., Lynfield, R., Lindegren, M. L., Bresee, J. & Finelli, L. The US Influenza Hospitalization Surveillance Network. *Emerg Infect Dis* **21**, 1543-1550 (2015). https://doi.org/10.3201/eid2109.141912
27 Matias, G. *et al.* Estimates of hospitalization attributable to influenza and RSV in the US during 1997-2009, by age and risk status. *BMC Public Health* **17**, 271 (2017). https://doi.org/10.1186/s12889-017-4177-z
28 Hung, H. M. *et al.* Molecular epidemiology and clinical features of rhinovirus infections among hospitalized patients in a medical center in Taiwan. *J Microbiol Immunol Infect* **52**, 233-241 (2019). https://doi.org/10.1016/j.jmii.2018.08.009
29 Akello, J. O. *et al.* Epidemiology of Human Adenoviruses: A 20-Year Retrospective Observational Study in Hospitalized Patients in Bern, Switzerland. *Clin Epidemiol* **12**, 353-366 (2020). https://doi.org/10.2147/CLEP.S246352
30 Nickbakhsh, S. *et al.* Epidemiology of Seasonal Coronaviruses: Establishing the Context for the Emergence of Coronavirus Disease 2019. *J Infect Dis* **222**, 17-25 (2020). https://doi.org/10.1093/infdis/jiaa185
31 Barrera-Badillo, G. *et al.* Human Metapneumovirus: Etiological Agent of Severe Acute Respiratory Infections in Hospitalized and Deceased Patients with a Negative Diagnosis of Influenza. *Pathogens* **9** (2020). https://doi.org/10.3390/pathogens9020085
32 Zhao, H., Harris, R. J., Ellis, J., Donati, M. & Pebody, R. G. Epidemiology of parainfluenza infection in England and Wales, 1998-2013: any evidence of change? *Epidemiol Infect* **145**, 1210-1220 (2017). https://doi.org/10.1017/S095026881600323X
33 Xin, H. *et al.* The Incubation Period Distribution of Coronavirus Disease 2019: A Systematic Review and Meta-analysis. *Clin Infect Dis* **73**, 2344-2352 (2021). https://doi.org/10.1093/cid/ciab501
34 Ganyani, T. *et al.* Estimating the generation interval for coronavirus disease (COVID-19) based on symptom onset data, March 2020. *Euro Surveill* **25** (2020). https://doi.org/10.2807/1560-7917.ES.2020.25.17.2000257
35 Park, M., Cook, A. R., Lim, J. T., Sun, Y. & Dickens, B. L. A Systematic Review of COVID-19 Epidemiology Based on Current Evidence. *J Clin Med* **9** (2020). https://doi.org/10.3390/jcm9040967
36 Spencer, J. A. *et al.* Distinguishing viruses responsible for influenza-like illness. *J Theor Biol* **545**, 111145 (2022). https://doi.org/10.1016/j.jtbi.2022.111145
37 Lessler, J. *et al.* Incubation periods of acute respiratory viral infections: a systematic review. *Lancet Infect Dis* **9**, 291-300 (2009). https://doi.org/10.1016/s1473-3099(09)70069-6
38 Cowling, B. J., Fang, V. J., Riley, S., Malik Peiris, J. S. & Leung, G. M. Estimation of the serial interval of influenza. *Epidemiology* **20**, 344-347 (2009). https://doi.org/10.1097/EDE.0b013e31819d1092
39 Biggerstaff, M., Cauchemez, S., Reed, C., Gambhir, M. & Finelli, L. Estimates of the reproduction number for seasonal, pandemic, and zoonotic influenza: a systematic review of the literature. *BMC Infectious Diseases* **14**, 480 (2014). https://doi.org/10.1186/1471-2334-14-480
40 Crowcroft, N. S. *et al.* Respiratory syncytial virus infection in infants admitted to paediatric intensive care units in London, and in their families. *Eur J Pediatr* **167**, 395-399 (2008). https://doi.org/10.1007/s00431-007-0509-9
41 Reis, J. & Shaman, J. Simulation of four respiratory viruses and inference of epidemiological parameters. *Infect Dis Model* **3**, 23-34 (2018). https://doi.org/10.1016/j.idm.2018.03.006
42 Poletti, P. *et al.* Evaluating vaccination strategies for reducing infant respiratory syncytial virus infection in low-income settings. *BMC Med* **13**, 49 (2015). https://doi.org/10.1186/s12916-015-0283-x
43 Matsuzaki, Y. *et al.* Human metapneumovirus infection among family members. *Epidemiol Infect* **141**, 827-832 (2013). https://doi.org/10.1017/S095026881200129X
44 Foy, H. M., Cooney, M. K., Hall, C., Malmgren, J. & Fox, J. P. Case-to-Case Intervals of Rhinovirus and Influenza Virus Infections in Households. *The Journal of Infectious Diseases* **157**, 180-182 (1988). https://doi.org/10.1093/infdis/157.1.180

45 Guo, Z. *et al.* Epidemiological analysis of an outbreak of an adenovirus type 7 infection in a boot camp in China. *PLoS One* **15**, e0232948 (2020). https://doi.org/10.1371/journal.pone.0232948
