# Supplementary material for: Impacts of human mobility on the citywide transmission dynamics of 18 respiratory viruses in pre- and post-COVID-19 pandemic years
Source: Nat Commun. 2024 May 16;15:4164. doi: 10.1038/s41467-024-48528-2 (PMC11098821; doi:10.1038/s41467-024-48528-2)
Supplement: Supplementary file 3 — Reporting Summary [file 41467_2024_48528_MOESM3_ESM.pdf]

nature portfolio

Corresponding author(s): Amanda C. Perofsky

Last updated by author(s): Apr 23, 2024

Reporting Summary

Nature Portfolio wishes to improve the reproducibility of the work that we publish. This form provides structure for consistency and transparency in reporting. For further information on Nature Portfolio policies, see our Editorial Policies and the Editorial Policy Checklist.

Statistics

For all statistical analyses, confirm that the following items are present in the figure legend, table legend, main text, or Methods section.

n/a	Confirmed	
☐	☒	The exact sample size (*n*) for each experimental group/condition, given as a discrete number and unit of measurement
☐	☒	A statement on whether measurements were taken from distinct samples or whether the same sample was measured repeatedly
☐	☒	The statistical test(s) used AND whether they are one- or two-sided<br>*Only common tests should be described solely by name; describe more complex techniques in the Methods section.*
☐	☒	A description of all covariates tested
☐	☒	A description of any assumptions or corrections, such as tests of normality and adjustment for multiple comparisons
☐	☒	A full description of the statistical parameters including central tendency (e.g. means) or other basic estimates (e.g. regression coefficient) AND variation (e.g. standard deviation) or associated estimates of uncertainty (e.g. confidence intervals)
☐	☒	For null hypothesis testing, the test statistic (e.g. *F*, *t*, *r*) with confidence intervals, effect sizes, degrees of freedom and *P* value noted<br>*Give P values as exact values whenever suitable.*
☐	☒	For Bayesian analysis, information on the choice of priors and Markov chain Monte Carlo settings
☒	☐	For hierarchical and complex designs, identification of the appropriate level for tests and full reporting of outcomes
☐	☒	Estimates of effect sizes (e.g. Cohen's *d*, Pearson's *r*), indicating how they were calculated

Our web collection on statistics for biologists contains articles on many of the points above.

Software and code

Policy information about availability of computer code

Data collection

No software was used to generate data for this study. PHI from study participants and patients contributing residual samples were collected through REDCap projects built by the Institute of Translational Health Sciences (ITHS).

Data analysis

Data processing and statistical analyses were performed with the statistical computing software R versions 4.3, unless otherwise noted. We used the National Institutes of Health's Biowulf HPC cluster (https://hpc.nih.gov/) to fit transmission models and perform block bootstrapping of time series cross-correlations. Biowulf used R version 4.2 at the time the code was originally written.

Code to reproduce the results and figures in this study are available at https://doi.org/10.5281/zenodo.11044821 and https://github.com/aperofsky/seattle_mobility_rt.

R packages used in this study:

Package management: renv (1.0.5)

Reading data and data manipulation: data.table (1.15.2), dplyr (1.1.4), dtplyr (1.3.1), forcats (1.0.0), magicfor (0.1.0), openxlsx (4.2.5.2), plyr (1.8.9), purrr (1.0.2), readr (2.1.5), reshape (0.8.9), tibble (3.2.1), tidyr (1.3.1), tidyverse (2.0.0)

String manipulation: glue (1.7.0), stringr (1.5.1)

Processing and analyzing SafeGraph data: SafeGraphR (0.5.2)

Epidemiological, survey, and mobility data sources: cdcfluview (0.9.4), covidcast (0.5.2)

Time series data manipulation and analysis: fasttime (1.1-0), forecast (8.22.0), lubridate (1.9.3), padr (0.6.2), tibbletime (0.1.8), tidyquant (1.0.7), timeDate (4032.109), timetk (2.9.0), tseries (0.10-55), TTR (0.24.3), xts (0.13.1), zoo (1.8-12)

Census data and spatial mapping: censusapi (0.8.0), sf (1.0-16), tidycensus (1.6.2), tigris (2.1)

Network analysis: igraph (2.0.3)

Epidemic modeling: epidemia (1.0.0), EpiEstim (2.2-4), EpiNow2 (1.4.0), R0 (1.3-1)

Statistical analysis, modeling, model comparison, and model performance: boot (1.3-30), boot.pval (0.5), caret (6.0-94), glmnet (4.1-8), gmodels (2.19.1), lognorm (0.1.10), Metrics (0.1.4), mgcv (1.9-1), MLmetrics (1.1.1), MuMIn (1.47.5), nlme (3.1-164), performance (0.10.9), tidymodels (1.1.1)

Parallelization: parallel (4.3.1), parallelly (1.37.1)

Visualization: ggplot2 (3.5.0), cowplot (1.1.3), ggExtra (0.10.1), gghighlight (0.4.1), ggpubfigs (0.0.1), ggpubr (0.6.0), gratia (0.8.2), jcolors (0.0.5), lattice (0.21-8), pals (1.8), patchwork (1.2.0), RColorBrewer (1.1-3), scales (1.3.0), viridis (0.6.5), viridisLite (0.4.2)

For manuscripts utilizing custom algorithms or software that are central to the research but not yet described in published literature, software must be made available to editors and reviewers. We strongly encourage code deposition in a community repository (e.g. GitHub). See the Nature Portfolio guidelines for submitting code & software for further information.

Data

Policy information about availability of data

All manuscripts must include a data availability statement. This statement should provide the following information, where applicable:

- Accession codes, unique identifiers, or web links for publicly available datasets
- A description of any restrictions on data availability
- For clinical datasets or third party data, please ensure that the statement adheres to our policy

Aggregated epidemiological and mobility data that support the findings of this study are available in a GitHub repository (https://github.com/aperofsky/seattle_mobility_Rt).

Access to deidentified individual-level study participant data requires a signed data access agreement with the Seattle Flu Alliance and can be made available to researchers whose proposed use of the data is approved by study investigators. Requests for data access should be submitted to data@seattleflu.org. Some mobility metrics were generated using SafeGraph Weekly Patterns and Social Distancing datasets, which were originally made freely available to academics in response to the COVID-19 pandemic. The SafeGraph Weekly Patterns dataset is currently available to academics for non-commercial use through an institutional university subscription or individual subscription to Dewey (https://www.deweydata.io/). The data access agreement with Dewey does not permit sharing of the raw data. Mobility data from Meta Data for Good Movement Range Maps are publicly accessible through the Humanitarian Data Exchange (https://data.humdata.org/dataset/movement-range-maps). SafeGraph social distancing data and Meta Data for Good survey data on masking are publicly accessible through the Carnegie Mellon Delphi group's COVIDcast Epidata API (https://cmu-delphi.github.io/delphi-epidata/api/covidcast.html). Data on the stringency of non-pharmaceutical interventions in US states are publicly accessible through the Oxford COVID-19 Government Response Tracker (https://github.com/OxCGRT/covid-policy-tracker).

Aggregated influenza syndromic and virologic surveillance data for Washington state are publicly accessible through the US Centers for Disease Control and Prevention (CDC) FluView Interactive dashboard (https://www.cdc.gov/flu/weekly/fluviewinteractive.htm). Aggregated respiratory syndromic surveillance data for King County, WA are not publicly available and were provided by the Rapid Health Information Network (RHINO) program at the Washington Department of Health (WA DOH). Access for research purposes requires a signed data sharing agreement with WA DOH and exemption approval from the Washington State Institutional Review Board. Requests for data access should be submitted to RHINO@doh.wa.gov. Data on COVID-19 cases in King County, WA are publicly accessible through the WA DOH COVID-19 dashboard (https://doh.wa.gov/emergencies/covid-19/data-dashboard). Data on COVID-19 vaccination in King County, WA are publicly accessible through the Public Health – Seattle & King County COVID-19 Vaccination dashboard (https://kingcounty.gov/en/dept/dph/health-safety/disease-illness/covid-19/data/vaccination). Nextstrain-curated SARS-CoV-2 sequence metadata can be downloaded via the Nextstrain CLI tool (https://docs.nextstrain.org/projects/cli/en/stable/).

Daily records of precipitation, temperature, and humidity in Seattle, WA are publicly accessible through the National Centers for Environmental Information’s U.S. Local Climatological Database (https://www.ncei.noaa.gov/products/land-based-station/local-climatological-data).

Research involving human participants, their data, or biological material

Policy information about studies with human participants or human data. See also policy information about sex, gender (identity/presentation), and sexual orientation and race, ethnicity and racism.

Reporting on sex and gender	The sex of study participants was self-reported at the time of enrollment in the Seattle Flu Study (SFS) or Seattle Coronavirus Assessment Network (SCAN) study. The self-reported sex of individuals tested at King County COVID-19 drive through testing sites was provided by Public Health - Seattle & King County. The sex of patients contributing clinically obtained specimens was provided by our hospital partners via electronic health records. However, sex was not used in the present study as a unit of analysis and was not considered when cleaning or filtering the individual-level surveillance data prior to analysis.<br><br>Sex information was not available for the aggregated and anonymous syndromic respiratory surveillance data obtained from US CDC and WA DOH. The human mobility data are aggregated and anonymous and only include information on home census block group (in aggregate) for mobile device users; thus, the sex of individuals visiting points of interest is not

available for those data.

Reporting on race, ethnicity, or other socially relevant groupings	The race and ethnicity of study participants were self-reported at the time of enrollment in the Seattle Flu Study (SFS) or Seattle Coronavirus Assessment Network (SCAN) study. The self-reported race and ethnicity of individuals tested at King County COVID-19 drive through testing sites were provided by Public Health - Seattle & King County. The race and ethnicity of patients contributing clinically obtained specimens were provided by our hospital partners via electronic health records. However, race and ethnicity are not included in the present study as units of analysis and were not considered when cleaning or filtering the individual-level surveillance data prior to analysis.<br><br>Race and ethnicity information were not available for the aggregated and anonymous syndromic respiratory surveillance data obtained from US CDC and WA DOH. The human mobility data are aggregated and anonymous and only include information on home census block group (in aggregate) for mobile device users; thus, the race/ethnicity of individuals visiting points of interest is not available for those data.
Population characteristics	For this study, we limited respiratory specimens to those collected from individuals residing in the greater Seattle region. Age group (<5 and >= 5 years of age) was considered when reconstructing daily pathogen incidences to adjust for discrepancies between the observed age distribution of positive specimens and the expected age distribution of cases in the community (derived from external data sources or published literature; see Methods in main text and Table S6 for more details).<br><br>The distributions of age groups and general home residence locations of the study population are reported in Table 1 in the main text of the manuscript. Regarding the age distribution of the entire study population, 84% of individuals were older than 4 years old and 70% were older than 18 years old. 81% of the study population resided in King County, WA, and the remaining individuals resided in other counties in WA state within the greater Seattle metropolitan area (Pierce, Snohomish, Kitsap, San Juan, Whatcom, Skagit, Island, Clallam, Jefferson, Mason, and Thurston counties). The distribution of age groups and home residence locations for different surveillance arms (hospital, SFS/SCAN community surveillance, and King County COVID-19 drive through testing sites) are provided in Table 1.
Recruitment	SFS and SCAN study participants were recruited via online recruitment, referrals from healthcare providers, targeted advertising via social media, word-of-mouth, earned and paid mass media, flyers posted at community locations, and direct recruitment by distributing priority codes to enable specific underrepresented populations to directly enroll. This included children, communities of color, community health center patients, essential workers, and individuals identified by contact tracing. With the exception of children, specimens from individuals recruited via priority codes were excluded from this study. There may have been biases in the individuals who agreed to participate in SFS or SCAN (see Hansen et al. JAMA Network Open 2022). Eligibility criteria changed over time in response to testing demand and and were based on Public Use Microdata Areas (PUMA) and reported symptoms. Each PUMA had a daily allocation of enrollments, with over sampling of PUMAs in southern King County to ensure more equitable access to testing across the county population.<br><br>Residual ("leftover") samples from clinically obtained specimens were used from patients who accessed healthcare. There was no participant interaction for this part of the study. These samples were collected on a regular basis from participating hospitals and medical systems, including UW Medical Center, Northwest Hospital, Harborview Hospital, Public Health – Seattle & King County, and Seattle Children's Hospital.
Ethics oversight	The Seattle Flu Study and Greater Seattle Coronavirus Assessment Network were approved by the Institutional Review Board of the University of Washington (protocols #00006181 and #000010432). At the time of enrollment, participants provided informed consent for respiratory sample and metadata collection and for the secondary use, banking and/or future sharing of de-identified data for research purposes. These IRB protocols explicitly approve the use of the surveillance data for secondary research and do not impose restrictions on the specific types of secondary research that can be conducted or the external data sources that can be analyzed in tandem with the surveillance data. In accordance with UW IRB approval, informed consent for residual sample and clinical data collection was waived, as these samples were already collected as part of routine clinical care, and it was not possible to re-contact these individuals. IRB exemption for the use of non-publicly available aggregated respiratory syndromic surveillance data for King County, WA was approved by the Washington State Institutional Review Board (Exempt Determination #2022-004). The human cellphone mobility data are aggregated and anonymous and were freely available to academic researchers prior to the start of this study; thus, these data do not constitute human subjects research. We did not collect cellphone data from surveillance study participants, and there is no individual-level linkage between the mobility and surveillance datasets. Individual-level linkage between these two datasets is not possible, given that the mobility data are aggregated and anonymous/de-identified. All other data sources pertaining to humans are aggregated, anonymous, and openly available. This study followed the Strengthening the Reporting of Observational Studies in Epidemiology (STROBE) reporting guidelines for cross-sectional studies.

Note that full information on the approval of the study protocol must also be provided in the manuscript.

Field-specific reporting

Please select the one below that is the best fit for your research. If you are not sure, read the appropriate sections before making your selection.

☒ Life sciences ☐ Behavioural & social sciences ☐ Ecological, evolutionary & environmental sciences

For a reference copy of the document with all sections, see nature.com/documents/nr-reporting-summary-flat.pdf

Life sciences study design

All studies must disclose on these points even when the disclosure is negative.

Sample size	In total, 138,050 respiratory specimens were screened for the presence of 24 or 26 pathogens, and we retained 80,891 specimens after

Sample size	limiting our analysis to individuals who were symptomatic for respiratory illness and discarding samples with missing metadata or from multiple testing. 25.5% (N = 20,659) of samples were collected in hospitals, and 74.5% (N = 60,232) were collected through community-based testing, including outpatient clinics, kiosks stationed in high foot traffic areas, swab-and-send at-home testing programs, and Public Health – Seattle & King County COVID-19 drive through testing sites. Sample sizes for each category of surveillance arm (hospital, SFS/SCAN community surveillance, King County drive through testing sites), disaggregated by home residence location and age group, are provided in Table 1 in the main text.<br><br>Sample sizes were not determined prior to the start of the study. While sampling was robust enough to provide granular (daily) surveillance data on the circulation of multiple pathogens, the diversity of sampling schemes required pre-processing to infer pathogen incidence. To properly reconstruct pathogen incidences through time, we considered the different populations sampled by SFS and SCAN, particularly regarding age group, clinical setting, and the presence of respiratory symptoms. To estimate incidence, daily PCR presence/absence data were adjusted for test volume, age, clinical setting (community or hospital), and local syndromic respiratory illness rates.
Data exclusions	In the methods section, we provide details concerning data exclusions under the "Reconstructing pathogen incidences" section. We first excluded samples with missing age or home address information (as reported by individuals participating in community surveillance or obtained through electronic hospital records), samples from individuals residing outside the greater Seattle region (King, Pierce, Snohomish, Kitsap, San Juan, Whatcom, Skagit, Island, Clallam, Jefferson, Mason, and Thurston counties), samples from individuals who were asymptomatic for respiratory illness, and samples from multiple testing of individuals. If an individual tested more than once in a 30-day period, we kept one result per pathogen in that period. If test results for all pathogens were consistent across the testing instances in the 30-day period, we kept the results from the first testing instance and discarded the subsequent instances. If an individual tested negative and then positive, or tested positive then negative, we kept the result for the first positive testing instance and discarded the instances prior to or after that result. We also excluded samples collected as part of Public Health – Seattle & King County's (PHSKC) contact tracing efforts or through collaborations with community-based organizations. Details for determining the respiratory symptom status of individuals from different surveillance arms are described in the Supplementary methods.<br><br>Out of the 26 pathogens included in the OpenArray panel, we limited our study to 18 pathogens for which sample sizes were sufficient to calculate daily incidences (see methods for inclusion criteria). We opted to estimate daily SARS-CoV-2 incidence from publicly available COVID-19 case data for King County instead of the OpenArray RT-qPCR data because the SCAN study did not test respiratory specimens for SARS-CoV-2 during May and June 2020.<br><br>We excluded hBoV, hPeV, MPn, and CPn from downstream analysis because probes for these pathogens were removed from our custom OpenArray panel in 2020. We also excluded ICV, EV.D68, measles, and mumps because these pathogens did not have a sufficient number of positive specimens to estimate daily incidence (< 200 positives from 2018 to 2022). Although Streptococcus pneumoniae was prevalent in samples collected prior to and during the COVID-19 pandemic, we did not include it in downstream analyses due to our inability to distinguish chronic carriage from acute infection.
Replication	Each respiratory specimen was screened in duplicate for a panel of respiratory pathogens using a custom TaqMan RT-qPCR OpenArray panel (Thermo Fisher). Laboratory methods are described in detail elsewhere (Hansen et al. JAMA Network Open 2022; Kim et al. J Clin Microbiol 2021). For a specimen to be designated positive for a given target, both duplicate samples must be positive in replicate RT-qPCR assays. More information about the TaqMan OpenArray assay can be found at https://assets.thermofisher.com/TFS-Assets/GSD/Application-Notes/openarray-respiratory-microbiota-taqman-app-note.pdf. Primer sequences cannot be shared because they are proprietary to Thermo Fisher.<br><br>We used nonparametric bootstrap tests with 1000 samples to estimate changes in pathogen Rt before and after two major events in our study: a major snowstorm in February 2019 and COVID-19 stay-at-home orders in March 2020.<br><br>We developed an ad-hoc test to determine the statistical significance of rolling window cross-correlations between mobility indicators and the daily transmission (Rt) of each pathogen. For each mobility indicator and pathogen pair in each rolling window, we used a block bootstrap approach to generate 1000 samples of the mobility time series shuffled in two week increments and recomputed weighted cross-correlations between Rt and mobility for each replicate, yielding a null distribution of 1000 cross-correlations. We considered cross-correlations between Rt and mobility indicators to be significant when observed coefficients were outside the bounds of the null distribution's 90% interval.
Randomization	There was no randomization because our study did not entail assigning specimens to experimental groups and control groups.
Blinding	The investigator who compiled the dataset and performed analyses for this study did not have access to PHI of study participants. Members of the lab and informatics teams had access to direct identifiers of biologic samples as well as extracted metadata, but staff maintained the minimum level of access required to complete their jobs. Otherwise, there was no blinding in the traditional sense because our study did not entail comparisons between experimental and control groups.

Reporting for specific materials, systems and methods

We require information from authors about some types of materials, experimental systems and methods used in many studies. Here, indicate whether each material, system or method listed is relevant to your study. If you are not sure if a list item applies to your research, read the appropriate section before selecting a response.

Materials & experimental systems

n/a	Involved in the study
☒	☐ Antibodies
☒	☐ Eukaryotic cell lines
☒	☐ Palaeontology and archaeology
☒	☐ Animals and other organisms
☒	☐ Clinical data
☒	☐ Dual use research of concern
☒	☐ Plants

Methods

n/a	Involved in the study
☒	☐ ChIP-seq
☒	☐ Flow cytometry
☒	☐ MRI-based neuroimaging

Plants

Seed stocks	*Report on the source of all seed stocks or other plant material used. If applicable, state the seed stock centre and catalogue number. If plant specimens were collected from the field, describe the collection location, date and sampling procedures.*
Novel plant genotypes	*Describe the methods by which all novel plant genotypes were produced. This includes those generated by transgenic approaches, gene editing, chemical/radiation-based mutagenesis and hybridization. For transgenic lines, describe the transformation method, the number of independent lines analyzed and the generation upon which experiments were performed. For gene-edited lines, describe the editor used, the endogenous sequence targeted for editing, the targeting guide RNA sequence (if applicable) and how the editor was applied.*
Authentication	*Describe any authentication procedures for each seed stock used or novel genotype generated. Describe any experiments used to assess the effect of a mutation and, where applicable, how potential secondary effects (e.g. second site T-DNA insertions, mosiacism, off-target gene editing) were examined.*
